# Exploring nonlinear and interaction effects of urban campus built environments on exercise walking using crowdsourced data

**DOI:** 10.3389/fpubh.2025.1549786

**Published:** 2025-01-30

**Authors:** Bo Lu, Qingyun Liu, Hao Liu, Tianxiang Long

**Affiliations:** ^1^School of Architecture and Art, Central South University, Changsha, China; ^2^Key Laboratory of Urban Planning Information Technology of Hunan Provincial Universities, Hunan City University, Yiyang, China; ^3^School of Geosciences and Info-Physics, Central South University, Changsha, China; ^4^College of Architecture and Urban Planning, Hunan City University, Yiyang, China; ^5^Key Laboratory of Digital Urban and Rural Spatial Planning of Hunan Province, Hunan City University, Yiyang, China

**Keywords:** exercise walking, university campus, machine learning, nonlinear relationships, interaction effects

## Abstract

**Introduction:**

University campuses, with their abundant natural resources and sports facilities, are essential in promoting walking activities among students, faculty, and nearby communities. However, the mechanisms through which campus environments influence walking activities remain insufficiently understood. This study examines universities in Wuhan, China, using crowdsourced data and machine learning methods to analyze the nonlinear and interactive effects of campus built environments on exercise walking.

**Methods:**

This study utilized crowdsourced exercise walking data and incorporated diverse campus characteristics to construct a multidimensional variable system. By applying the XGBoost algorithm and SHAP (SHapley Additive exPlanations), an explainable machine learning framework was established to evaluate the importance of various factors, explore the nonlinear relationships between variables and walking activity, and analyze the interaction effects among these variables.

**Results:**

The findings underscore the significant impact of several key factors, including the proportion of sports land, proximity to water bodies, and Normalized Difference Vegetation Index NDVI, alongside the notable influence of six distinct campus area types. The analysis of nonlinear effects revealed distinct thresholds and patterns of influence that differ from other urban environments, with some variables exhibiting fluctuated or U-shaped effects. Additionally, strong interactions were identified among variable combinations, highlighting the synergistic impact of elements like sports facilities, green spaces, and waterfront areas when strategically integrated.

**Conclusion:**

This research contributes to the understanding of how campus built environments affect walking activities, offering targeted recommendations for campus planning and design. Recommendations include optimizing the spatial configuration of sports facilities, green spaces, and water bodies to maximize their synergistic impacts on walking activity. These insights can foster the development of inclusive, health-promoting, and sustainable campuses.

## 1 Introduction

Walking, as the most fundamental and universal mode of transportation and a form of light physical activity, serves as a primary means of green and low-carbon urban travel (1, 2). Studies have demonstrated that walking effectively reduces the incidence of non-communicable diseases, such as obesity, and provides significant social benefits (3, 4). University campuses, as integral components of cities, possess spacious and picturesque environments, minimal exposure to urban traffic, and abundant exercise facilities (e.g., pathways, and sports fields). These attributes facilitates walking, jogging, and other physical activities, fostering active lifestyles (5, 6).

Traditionally, Chinese university campuses differ from their international counterparts due to their closed management systems and perimeter walls. However, with the implementation of the “Open Campus” policy (7) has made the campus environments more accessible to the public. This shift alleviates the shortage of amenities in nearby urban communities and promotes integration between campus and city environments, leading to shared utilization of the campus resources.

Scholars have established theoretical frameworks and methodologies to explore the relationship between urban built environment and behavioral activities. These studies examined the impact of campus built environments on walking behavior from multiple perspectives (8, 9), revealing that campus design and planning can significantly promote or inhibit walking activities (10, 11). Existing researches primarily focus on the walkability of university campuses and investigates how various campus built environments influence students’ travel behavior, willingness to travel, and overall health (8, 9, 12). Methodologically, scholars have predominantly rely on audits and questionnaires, integrated with GIS spatial data and modified urban walkability measurement tools (e.g., NEWS-A (13), PACES (14)). Multivariate linear regression models, negative binomial regression models, and structural equation models are frequently used to evaluate the linear relationships between campus walking environments and influencing factors (15).

Research has shown that natural environment factors, service facility density, destination accessibility, and active transportation compatibility (e.g., intersections, road conditions, walking/biking capacity) are positively correlated with the intensity of walking activities in campus (9, 16, 17). Studies have also highlighted the role of proximity to exercise facilities on students’ walking activities. Reed found that closer proximity to sports venues promotes physical activities and increases individuals’ willingness to engage in exercise (18). Additionally, some scholars have emphasized the distinctions between subjective perceptions and objective assessments of campus environments in influencing walking activities (9).

Recent advancements in big data technology and machine learning have drawn attention to the nonlinear effects of urban built environments on human behavior. Studies employing machine learning techniques such as Gradient Boosting Decision Trees (GBDT) (19), Random Forests (20), and XGBoost (21) have been used to investigate the nonlinear relationships between built environments and factors like active travel, travel preferences, and walking intentions (22–24). These methods relax preset conditions, accommodate diverse data types, and offer higher predictive accuracy, enabling the precise capture of complex relationships between variables (25, 26).

Numerous studies have demonstrated that nonlinear relationships are prevalent between the built environments and physical activities (22, 23). For instance, Cheng et al. found that population density and land use diversity only promote walking within certain thresholds (27). Similarly, Zeng et al. revealed that variations in building density exhibit nonlinear effects on pedestrian traffic, with walking flow peaking at a building density of approximately 0.3 (28). Yang et al. combined a Random Forest model with geographically weighted regression (GWR) and used SHAP analysis to explore the nonlinear effects of built environment factors on jogging in Beijing, revealing varying influences of factors like population density, parks, and green landscape index across different contexts (20). These findings challenge the validity of widely assumed linear relationships, enabling a more precise understanding of variable interactions (29). Among these methods, researchers frequently combine machine learning with Partial Dependence Plots (PDPs) to effectively model high-dimensional data and uncover complex nonlinear relationships between features (30, 31).

Current researches on the impact of campus environments on walking activities has several limitations. First, data acquisition methods predominantly rely on “small data” approaches, such as cross-sectional surveys and on-site audits, to measure built environments (10, 12). Few studies leveraging “big data” approaches, employing crowdsourced data and quantitative evaluation tools across multiple scale, are relatively rare (21, 32). Second, existing research largely relies on research methods developed by Ewing et al. (33), using pedestrian flow rates from field surveys as primary indicators of walking activities intensity, with limited differentiation between commuting, recreational or exercise walking. However, different types of walking activities in different environments may be influenced by distinct factors (19, 34, 35). Lastly, methodologies in this field primarily use descriptive statistics and regression models to establish linear associations (32). While useful, such approaches are insufficient for accurately uncovering the patterns complex patterns, underestimating potential influences and ignoring nonlinear relationships and synergies between variables.

To address these research limitations, this study focuses on Wuhan, a city with a significant concentration of universities, as the case study area. The research focuses on evaluating and exploring the relationship between campus-built environments (CBEs) and exercise walking (EW), aiming to answer the following questions: (1) How to construct a multidimensional research framework tailored to campus environments? (2) Identifying relative importance CBE variables on influencing EW. (3) Whether CBE variables exhibit nonlinear and interaction effects on EW, and how do these effects manifest? Using crowdsourced data on EW routes from university campuses in Wuhan, involving diverse users such as faculty, students, and visitors, this study employs advanced tools, including ArcGIS, sDNA, and semantic segmentation models, to construct datasets characterizing campus environments. Guided by the “5D + S” (36) research framework, a multidimensional variable system was developed to capture essential CBE features. The study developed an interpretable machine learning regression model using XGBoost and SHAP model, addressing the underexplored complex nonlinear and interactive relationships between CBE and EW. This research provides valuable insights for policymakers, urban planners, and campus administrators, highlighting planning and design strategies to create healthy, inclusive, and sustainable campus environments that enhance EW participation and broader community health outcomes.

## 2 Materials and methods

### 2.1 Study area

**Figure 1:**
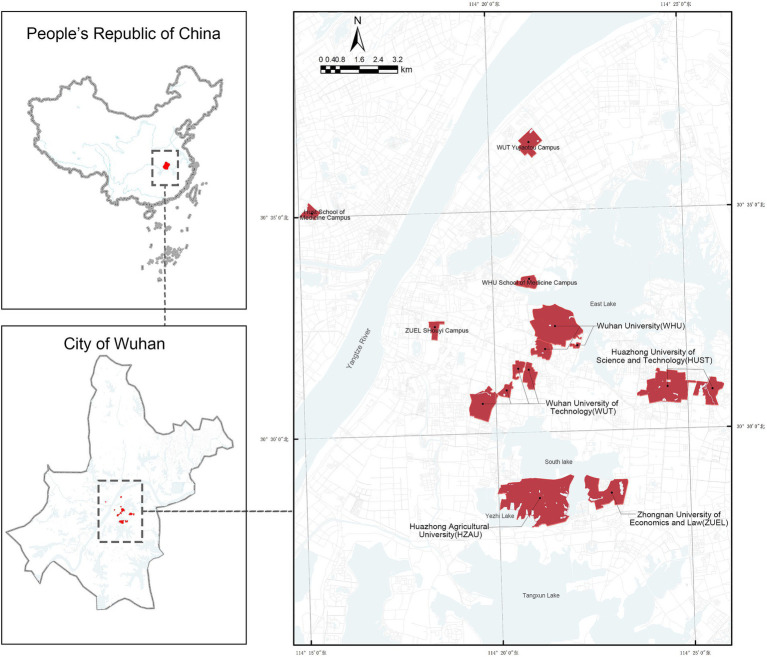
Distribution of the top five largest university campuses in Wuhan.

In this study, the five largest universities in Wuhan (30.5928° N, 114.3055°E)in terms of campus area were selected as research subjects: Wuhan University (WHU), Huazhong University of Science and Technology (HUST), Huazhong Agricultural University (HZAU), Zhongnan University of Economics and Law (ZUEL), Wuhan University of Technology (WUT) and their respective sub-campuses (Figure 1). As a major central city in Southern China, Wuhan is renowned for its strong higher education system, ranking third in the country with 83 universities. The city’s geographical features include an interwoven mix of plains and hills with numerous rivers and lakes. Campuses such as WHU, HUST and HZAU are bordered by large lakes, highlighting the importance of incorporating natural environmental elements into the research framework. Collectively, these universities enroll approximately 240,000 students and span a total area of about 18 km^2^. Their varied spatial scales, layouts, and geographical characteristics make them ideal subjects for this study, offering a robust basis for analyzing the impact of campus built environments on physical activity.

### 2.2 Data collection and analysis

The EW data used in this study was obtained from the Dorray fitness application, which categorizes various physical activities and provides key attributes such as spatial location, time, duration, and distance along with walking trajectory data. The study utilized raw walking data collected between 2017 and 2018 for two primary reasons: first, the COVID-19 pandemic significantly restricted walking activities in China from early 2020 through late 2022; second, the two-year period provided a larger dataset, allowing for more robust observations of EW variations.

Data preprocessing was conducted in ArcGIS 10.8. All EW data from the top 5 university campuses in Wuhan was selected, and invalid trajectories caused by anomalies, such as spatial shifts (<1 meter), short activity durations (<1 min), and low speeds (<4 km/h, as defined by the minimum walking speed), were excluded. After cleaning, 5,922 valid trajectories were retained. Following previous research cases (32, 37) and considering the smaller scale of this study, a 50 m × 50 m fishnet grid was created for the 10 campuses using the ArcGIS “Fishnet” tool, overlaying the walking trajectories. This process resulted in 3,557 grids containing EW trajectory data.

**Figure 2:**
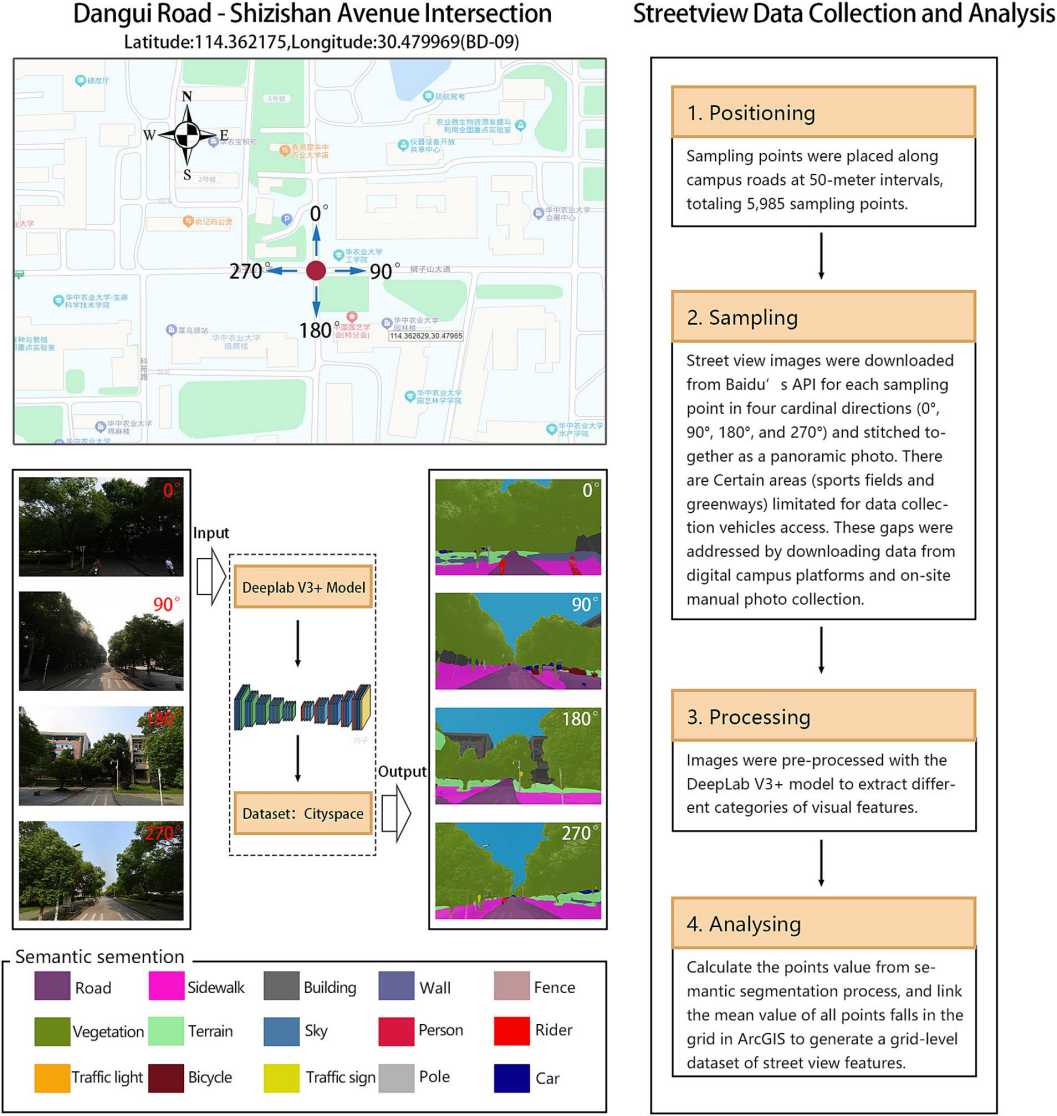
Street view sampling and semantic segmentation with DeepLab V3 + model.

Macroscale variables related to satellite-level of campus built environments data, including natural landscapes, urban roads, urban buildings, population density, and land use types, were sourced from the Chinese Resource and Environmental Science Data Center and open-source platforms such as OpenStreetMap. Data on infrastructure and public service points of interest (POI) was gathered from Gaode Maps, while NDVI (Normalized Difference Vegetation Index) data was retrieved from the National Tibetan Plateau Data Center. ArcGIS tools were used to aggregate and process these spatial datasets, and sDNA software was employed to analyze road network data, generating spatial structure indicators. Microscale variables related to eye-level street view data was mostly obtained from Baidu Panorama Map. We used DeepLab V3+ model and ArcGIS to process the streetview data (Figure 2). Processing the street view images, including semantic segmentation and post-processing, required approximately 12 h on a computer with Intel Core i7 CPU and NVIDIA GeForce GTX 1070 GPU. All the data were obtained between June 2017 to May 2018 to minimize temporal discrepancies with the walking trajectory data.

### 2.3 Variables

Based on previous research on walking indices (38, 39), the EW was calculated as the total number of cleaned walking trajectories within each grid using ArcGIS’s spatial join and data aggregation tools. The EW serves as the dependent variable in this study. Statistical analysis (Table 1) shows that the mean EW is relatively low, indicating a generally low frequency of walking activities within the regions.

**Table 1:** Descriptive statistics of variables.

Variable type	Variable name	Description	Min	Max	Mean	Std. Dev
Dependent variable	Exercise Walking (EW)	Total number of walking trajectories within a grid unit	1	140	12.83	18.70
POI accessibility	Distance to Public Transport (DT)	Nearest distance to public transport facilities (m)	2.17	880.52	173.65	151.91
	Distance to Fitness or leisure Facilities (DF)	Nearest distance to fitness or leisure facilities (m)	1.90	1121.46	210.14	170.49
	Distance to Scenic Spots (DL)	Nearest distance to scenic spots (m)	3.35	1509.27	337.51	275.34
	Distance to Daily Services Facilities (DP)	Nearest distance to daily service facilities (m)	0.44	779.59	121.74	102.73
	Distance to Dining Services Facilities (DC)	Nearest distance to dining facilities (m)	1.44	974.34	200.93	166.78
Density	Building Density (BD)	Proportion of land area occupied by buildings	0	0.98	0.13	0.16
	Road Density (RD)	Total road length within a grid unit (m)	0	295.27	61.82	45.56
	Population Density (PD)	Number of people per hectare	0	670.52	104.09	100.82
Land Use	Sports Land Use (SL)	Proportion of sports land within a grid unit	0	1	0.06	0.19
	Educational and Research Land Use (TL)	Proportion of educational and research land within a grid unit	0	1	0.32	0.39
	Residential Land Use Density (RL)	Proportion of residential land within a grid unit	0	1	0.23	0.36
	Land Use Entropy (LE)	Indicator of land use diversity	0	1.28	0.29	0.28
Spatial Network	Closeness Centrality (NQ)	Difficulty of access within a given radius	0	0.57	0.12	0.10
	Betweenness Centrality (BC)	Centrality of road segments	0	4.57	0.75	0.65
	Detour Ratio (DR)	Degree of detour within the road network	0	4.22	1.12	0.55
	Route Efficiency (RE)	Maximum coverage of road segments within a grid	0	408.11	168.56	83.68
	Block Scale (SB)	Ratio of total road length to the number of intersections	0	41.04	6.85	6.31
Natural Landscape	Proximity to Water Bodies (DW)	Nearest distance to water bodies (m)	0	1115.2	224.72	204.92
	Green Space Density (DG)	Proportion of green space within a grid unit	0	1	0.40	0.34
	Average Slope (SR)	Vertical to horizontal height ratio (%)	0	13.60	2.44	1.78
	Normalized Difference Vegetation Index (NDVI)	Average NDVI value of grid elements	−0.20	0.84	0.32	0.21
Street environment	Green View Index (GVI)	Percentage of vegetation pixels in an image	0	0.99	0.47	0.23
	Sky View Index (SVI)	Percentage of sky pixels in an image	0	0.74	0.26	0.21
	Vehicular Movement Index (VMI)	Percentage of motorized traffic pixels in an image	0	0.38	0.10	0.05
	Sidewalk Coverage Ratio (RS)	Percentage of sidewalk pixels relative to road surface	0	1	0.30	0.25
	Visual Humanization Index (VHI)	Percentage of human-centered design elements in an image	0	0.29	0.05	0.04
	Shannon Diversity Index (SDI)	Diversity and balance of spatial elements	0	0.99	0.63	0.14

The macroscale CBE variables system was constructed using the widely adopted “5D + S” research framework (15, 36). Given the distinct characteristics of campus environments compared to typical urban communities, the variable system integrates modifications from campus-specific walking environment studies (12, 32, 40). At the macroscale level, 21 environmental variables were selected, categorized into five groups: (1) Accessibility to Points of Interest (POI): Distance to public transport facilities (campus parking lots, bus stops) (DT), Distance to fitness or leisure facilities (small sports grounds, gyms, game rooms) (DF), Distance to scenic spots (campus squares, cultural sculptures, pocket parks) (DL), Distance to public service facilities (campus convenience stores, small supermarkets, eyewear shops, photo printing services) (DP), Distance to dining service facilities (restaurants, fast food outlets, bakeries) (DC). (2) Density variables: Building density (BD), Road density (RD), Population density (PD). (3) Building and land use density variables: Residential land use density (RL), Educational and research land use density (TL), Sports land use density (SL), Land use entropy (LE). (4) Spatial network structure variables: Closeness centrality (NQ), Betweenness centrality (BC), Detour ratio (DR), Route efficiency (RE), Block scale (SB). (5) Natural environmental variables: Proximity to water bodies (DW), Green space density (DG), Average slope (SR), Normalized difference vegetation index (NDVI).

At the microscale, six street-level visual factor variables were included based on previous research (20, 41): Green View Index (GVI), Sky View Index (SVI), Vehicular Movement Index (VMI), Visual Humanization Index (VHI), Sidewalk Coverage Ratio (RS), and Shannon Diversity Index (SDI).

The following equations illustrate how some of the variable values in the study are calculated. LE was derived from the Shannon entropy index, which measures the diversity of land use within a region (32). The formula is as follows (Equation 1):


(1) $$LE=-\frac{\sum_{1}^{n}P_{i}\ln P_{i}}{\ln\left(n\right)}$$

where $P_{i}$ represents the proportion of a specific land use type in the total area. n is the number of land use types.

NQ measures the connectivity of a network by calculating the ratio of network links to the Euclidean distances between an origin point and all accessible destinations within a given radius (32). The formula is as follows (Equation 2):


(2) $$NQ=\underset{y\in R_{x}}{\sum }\frac{P\left(y\right)}{dM\left(x,y\right)}$$

Where *R*_x_ represents the set of links starting from link x within a given network radius. $P\left(y\right)$ represents the weight of node y within the search radius. dM(x,y) is the shortest Euclidean distance from node x to node y (32).

BC is defined as the number of all possible trips through a network link. The formula is as follows (Equation 3):


(3) $$BC=\underset{y\in N}{\sum }\underset{y\in R_{x}}{\sum }P\left(z\right)OD\left(y,z,x\right)$$

where $OD\left(y,z,x\right)$ represents the geodesic distance between endpoints y and z that passes through link x (32).

DR quantifies the degree of detour within a network by calculating the average ratio of geodesic link lengths to crow-fly distances within a given radius. The formula is as follows (Equation 4):


(4) $$DR=\frac{\sum_{y\in R_{x}}\frac{dM\left(x,y\right)}{CFD\left(x,y\right)}W\left(y\right)P\left(y\right)}{\sum_{y\in R_{x}}W\left(y\right)P\left(y\right)}$$

where $CFD\left(x,y\right)$ represents the crow-fly distance between the centers of x and y (32, 36).

RE refers to the maximum radius of a convex hull within the network radius, reflecting the maximum spatial coverage of a network’s area (1, 42).

Among Microscale variables, GVI and SVI represent the percentages of vegetation pixels and sky pixels, respectively, in a given image. Both have been shown to significantly influence outdoor physical activity and subjective perceptions (41, 43, 44). The formulas are as follows (Equations 5, 6):


(5) $$GVI=\frac{P_{\mathrm{green}}}{P_{\mathrm{total}}}\times 100\%$$

(6) $$SVI=\frac{P_{\mathrm{sky}}}{P_{\mathrm{total}}}\times 100\%$$

where $P_{\mathrm{green}}$ represents the number of vegetation pixels in the image, $P_{\mathrm{sky}}$ represents the number of sky pixels in the image, and $P_{\mathrm{total}}$ represents the total number of pixels in the image.

VMI and VHI quantify the degree of motorization and humanization in street spaces, reflecting the dominance of vehicular elements versus pedestrian-friendly features (20, 44). Their formulas are as follows (Equations 7, 8):


(7) $$VMI=\overset{n}{\underset{1}{\sum }}\left(P_{\mathrm{road}}+P_{\mathrm{trafficlight}}+P_{\mathrm{trafficsign}}+P_{\mathrm{car}}\right)$$

(8) $$VHI=\overset{n}{\underset{1}{\sum }}\left(P_{\mathrm{person}}+P_{\mathrm{sidewalk}}+P_{\mathrm{rider}}+P_{\mathrm{bike}}\right)$$

where $P_{\mathrm{road}}$ , $P_{\mathrm{trafficlight}}$ , $P_{\mathrm{trafficsign}}$ , $P_{\mathrm{car}}$ represent pixels of roads, traffic lights, traffic signs, and cars in the image; $P_{\mathrm{person}}$ , $P_{\mathrm{sidewalk}}$ , $P_{\mathrm{rider}}$ , $P_{\mathrm{bike}}$ represent pixels of pedestrians, sidewalks, riders and bicycles in the image.

RS represents the proportion of sidewalk area relative to the total road surface area, reflecting the quality of street space. Higher RS values are positively associated with walking activities (40, 41). The formula is as follows (Equation 9):


(9) $$RS=\frac{P_{\mathrm{sidewalk}}}{P_{\mathrm{sidewalk}}+P_{\mathrm{road}}}$$

SDI describes the diversity and balance of visual spatial elements. A higher SDI indicates a more diverse and balanced spatial landscape, which has been proven to be both applicable and intuitive (21, 44). The formula is as follows (Equation 10):


(10) $$SDI=1-\overset{n}{\underset{1}{\sum }}{P_{i}}^{2}$$

where represents the proportion of pixels of element type i relative to the total pixels, and n represents the number of element types. After semantic segmentation, visual features were extracted using the DeepLab V3+ model.

Macroscale and microscale CBE variables were computed and are summarized (Table 1).

### 2.4 Methods

**Figure 3:**
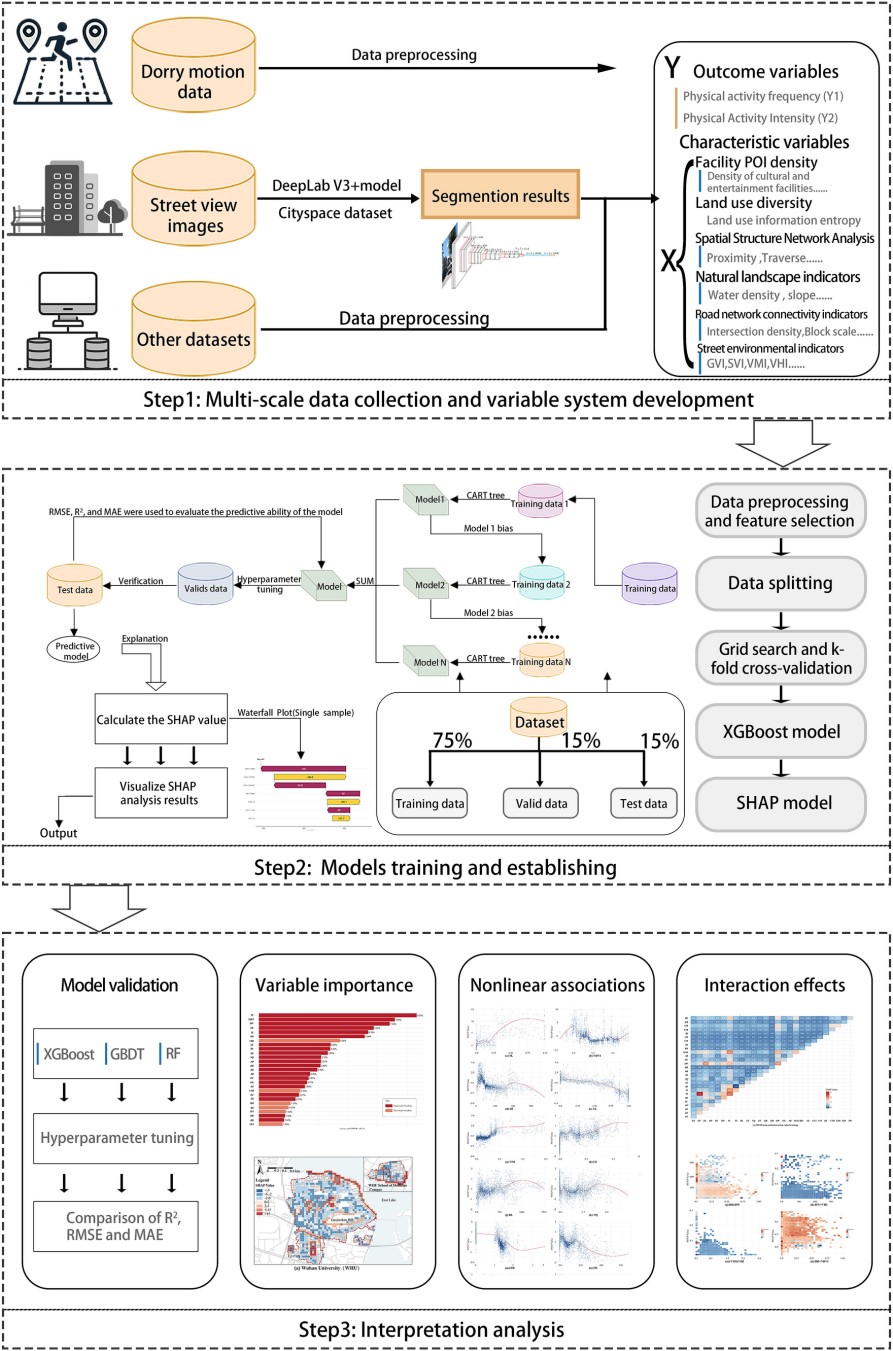
The proposed analytical workflow of the study.

The workflow of this study is divided into three main steps: First, data collection and variable system development: This step involves collecting and cleaning the data, followed by the establishment of the variable system. Secondly, XGBoost model training and establishing: This step focuses on implementing and training the model. The model training process utilizes several packages in R 4.4.2, including ‘XGBoost’ ‘caret’ ‘shapviz’ ‘shapr’. To address the “black-box” nature of the XGBoost model, SHAP (SHapley Additive exPlanations) was employed to analyze the model’s nonlinear interpretability. Thirdly, Interpretation analysis: This step includes validating the model’s performance and interpreting results such as feature importance (RI), nonlinear correlations, and interaction effects using SHAP. The training and hyperparameter tuning process for all models required approximately 10 h on a computer with Intel Core i7 CPU and NVIDIA GeForce GTX 1070 GPU (Figure 3).

This study employs the Extreme Gradient Boosting (XGBoost) regression tree model to examine the relationship between campus environments and walking activities. XGBoost, introduced by Chen and Guestrin (45), is an optimized distributed gradient boosting library based on Gradient Boosting Decision Trees (GBDT). It is designed for efficient, flexible, and portable machine learning models. By tuning hyperparameters, XGBoost can optimize performance and better explain complex nonlinear relationships among variables (25, 26). GBDT is often compared to another widely used ensemble method, Random Forest. While GBDT is based on the boosting technique, Random Forest employs bagging. In general, although GBDT models are typically more complex and time-consuming, they often outperform Random Forest in terms of accuracy (46).

In this study, we present the key equations of XGBoost to illustrate the theoretical foundation of this model as follows. Additional details can be found in the paper by Chen and Guestrin (45). XGBoost constructs an additive tree-based model where the prediction for each sample is obtained by summing the outputs of K regression trees. The predicted value ${\hat{y}}_{i}$ is expressed as (Equation 11):


(11) $${\hat{y}}_{i}=\overset{k}{\underset{K=1}{\sum }}f_{k}\left(X_{i}\right),f_{k}\in F$$

where $f_{k}$ represents the k-th tree. The objective function includes a loss term, measuring the difference between true and predicted values, and a regularization term, controlling model complexity. The objective function is defined as (Equation 12):


(12) $$L=\overset{n}{\underset{i=1}{\sum }}l\left(y_{i},{\hat{y}}_{i}\right)+\overset{K}{\underset{k=1}{\sum }}\Omega \left(f_{k}\right)$$

where $l\left({\hat{y}}_{i},y_{i}\right)$ represents loss function, measuring the difference between the true and predicted values. $\Omega \left(f_{k}\right)$ represents the regularization term, penalizing model complexity to prevent overfitting, defined as (Equation 13):


(13) $$\Omega \left(f_{k}\right)=\gamma Τ+\frac{1}{2}\lambda \overset{T_{k}}{\underset{j=1}{\sum }}w_{j}^{2}$$

where $T_{k}$ represents the number of leaves in the k-th tree, $w_{j}$ represents the weight of the j-th leaf, γ represents the penalty parameter controlling the number of leaves, and *λ* represents the regularization parameter controlling the magnitude of leaf weights.

XGBoost iteratively adds trees to the model using the gradient boosting method. At each iteration t, a new tree is added to minimize the following objective function (Equation 14):


(14) $$L^{\left(t\right)}\approx \overset{n}{\underset{i=1}{\sum }}\left[g_{i},f_{t}\left(x_{i}\right)+\frac{1}{2}h_{i}f_{t}{\left(x_{i}\right)}^{2}\right]+\Omega \left(f_{k}\right)$$

where $g_{i}$ and $h_{i}$ are the first and second order gradients of the loss function with respect to the predictions. These gradients are defined as (Equations 15, 16):


(15) $$g_{i}=-\frac{\partial l\left(y_{i},{\hat{y}}_{i}\right)}{\partial {\hat{y}}_{i}}$$

(16) $$h_{i}=-\frac{\partial^{2}l\left(y_{i},{\hat{y}}_{i}\right)}{\partial {\hat{y}}_{i}^{\left(2\right)}}$$

Node splitting is a critical step in tree construction. Splits are determined by maximizing the gain, which measures the improvement in the objective function. Each tree’s structure is determined by potential splits that maximize the gain (Equation 17):


(17) $$\mathrm{Gain}=\frac{1}{2}\left[\frac{G_{L}^{2}}{H_{L}+\lambda }+\frac{G_{R}^{2}}{H_{R}+\lambda }+\frac{{\left(G_{L}+G_{R}\right)}^{2}}{H_{R}+H_{L}+\lambda }\right]-\gamma$$

where $G_{L}$ and $G_{R}$ denote sum of first-order gradients for the left and right child nodes, $H_{R}$ and $H_{L}$ denote sum of second-order gradients for the left and right child nodes. The regularization parameter *γ* and *λ* can control the complexity of the tree to prevent overfitting.

To interpret the XGBoost model, this study applies SHAP values to interpret the XGBoost model. SHAP values, derived from game theory’s Shapley value concept, are a powerful tool for explaining machine learning model predictions (47). SHAP provides robust, scalable, and interpretable insights, helping to understand the contribution of each feature to model predictions, both globally and locally, particularly valuable for complex “black-box” models like XGBoost and neural networks (48). The SHAP value for a feature i is expressed as follows (Equation 18):


(18) $$\phi_{i}=\underset{S\subseteq N\backslash \left\{i\right\}}{\sum }\frac{|S|!\left(|N|-|S|-1\right)!}{|N|!}\left[f\left(S\cup \left\{i\right\}\right)-f\left(S\right)\right]$$

where $\phi_{i}$ represents the contribution of feature i, N represents the set of all p features, $f\left(S\cup \left\{i\right\}\right)$ and $f\left(S\right)$ represents model prediction with and without feature i, respectively. The SHAP value $\phi_{i}$ can be positive, negative, or zero, representing whether the feature increases, decreases, or does not affect the prediction, respectively. The absolute SHAP value reflects the magnitude of the feature’s impact on the model’s output. The relative importance of a feature is calculated as the average of its absolute SHAP values (Equation 19):


(19) $$f\left(x\right)=\phi_{0}+\overset{M}{\underset{i=1}{\sum }}\phi_{i}$$

where M represents number of input features, $\phi_{0}$ represents base value of the model output, and $\phi_{i}$ represents Shapley value for feature i. This approach allows for interpreting the contributions of individual features and understanding the nonlinear effects within the XGBoost model.

## 3 Results

### 3.1 Model validation

To validate the model more effectively, we compared the performance of three different machine learning models: the XGBoost, the GBDT, and the Random Forest regression model. To ensure consistency in experimental conditions, we first examined multicollinearity among the independent variables and removed variables with a Variance Inflation Factor (VIF) greater than 10 (20, 49, 50). Given our objective to compare model performance rather than deploy models on new data, and considering the relatively small sample size (3,557 grids), we divided the dataset into 80% training and 20% validation data. To analyze the data distribution of different variables across various datasets, we conducted a comprehensive assessment using the Kolmogorov–Smirnov (KS) test and histogram plots. The analytical results are available in the Supplementary materials. Our analysis revealed that most variables exhibited consistent distributions across datasets. However, the dependent variable (WE) showed skewness. To address this and improve data quality, we applied preprocessing techniques, including square root transformation and standardization, prior to conducting hyperparameter tuning.

To enhance model performance and mitigate overfitting, this study employed Bayesian optimization for systematic hyperparameter tuning of the XGBoost model. Bayesian optimization efficiently identifies optimal combinations of hyperparameters by leveraging a probabilistic model to guide the search process, focusing on promising regions of the hyperparameter space while minimizing the number of evaluations (51). Additionally, 5-fold cross-validation was utilized to ensure the robustness of the model. For reproducibility, key hyperparameters were optimized within the following ranges: learning rate (eta: 0.01–0.05) to control iteration step size; tree depth (max_depth: 6–10) for model complexity and nonlinear relationships; sample ratio (subsample: 0.6–0.9) to reduce overfitting by selecting subsets of training samples; feature sampling ratio (colsample_bytree: 0.6–0.9) for features per tree; minimum leaf weight (min_child_weight: 10–30) to enhance robustness; minimum split loss (gamma: 0–5) to prevent excessive splitting; and L2 (lambda) and L1 (alpha) regularization (1–20) to limit model complexity.

The optimization began with 10 initial points to construct the surrogate model, followed by 60 iterations to identify the optimal parameters. A similar preprocessing and tuning strategy was applied for GBDT and Random Forest, enabling a comparative analysis of predictive performance. The best XGBoost parameters were determined as follows: eta: 0.0328, max_depth: 10, subsample: 0.9, colsample_bytree: 0.7862, min_child_weight: 10, minimum split loss: 0, lambda: 20, alpha: 5.

The machine learning model’s performance is assessed primarily by its predictive power, which is commonly evaluated using three key metrics (20, 24): Coefficient of Determination (R^2^): Measures the goodness of fit for statistical models with values ranging from 0 to 1; higher values indicate better predictive accuracy. Root Mean Squared Error (RMSE): Represents the square root of the mean squared differences between predicted and actual values, with smaller values indicating better accuracy. Mean Absolute Error (MAE): The average of the absolute differences between predicted and actual values, with smaller values indicating higher precision.

The results (Table 2) highlight the core parameter metrics and performance of the three models. They indicate that the XGBoost model achieved a relatively higher R^2^ value than the other two models, while its RMSE and MAE values were lower. These findings demonstrate that the XGBoost model outperformed the GBDT and Random Forest models in this study, highlighting its enhanced capability to address nonlinear regression problems (46).

**Table 2:** Model parameters and performance evaluation.

Model	Parameters			Performance		
	N_estimators	Learning rate	Max_depth	RMSE	MAE	R^2^
XGboost	1,436	0.03281965	10	12.8558	7.1553	0.6206
GBDT	2,988	0.03907009	10	13.2035	7.5187	0.5654
Random Forest	3,000	/	8	15.3829	8.2768	0.4608

### 3.2 RI

**Figure 4:**
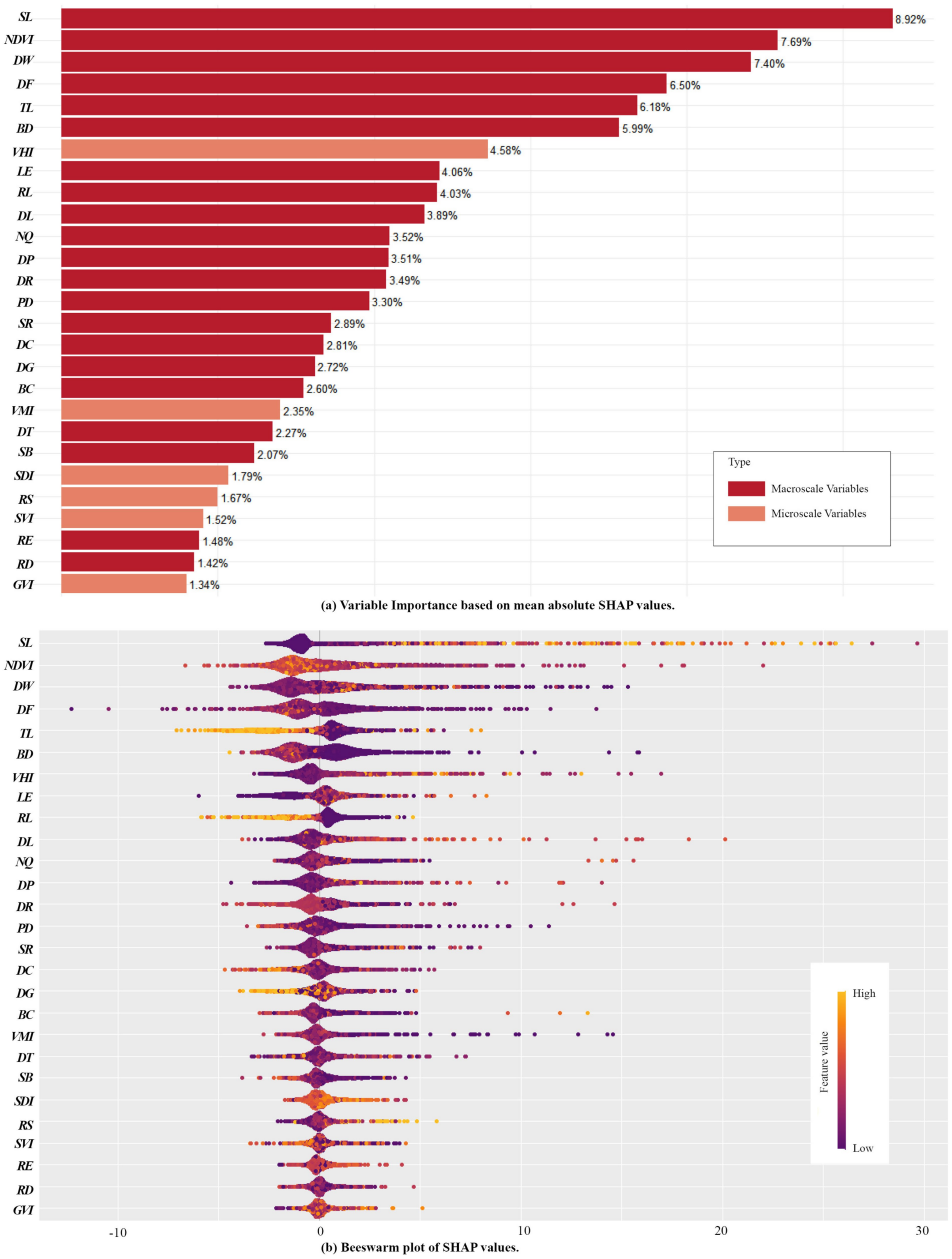
RI of Variables derived using the SHAP model. **(a)** Variable Importance based on mean absolute SHAP values; **(b)** Beeswarm plots of SHAP values.

**Figure 5:**
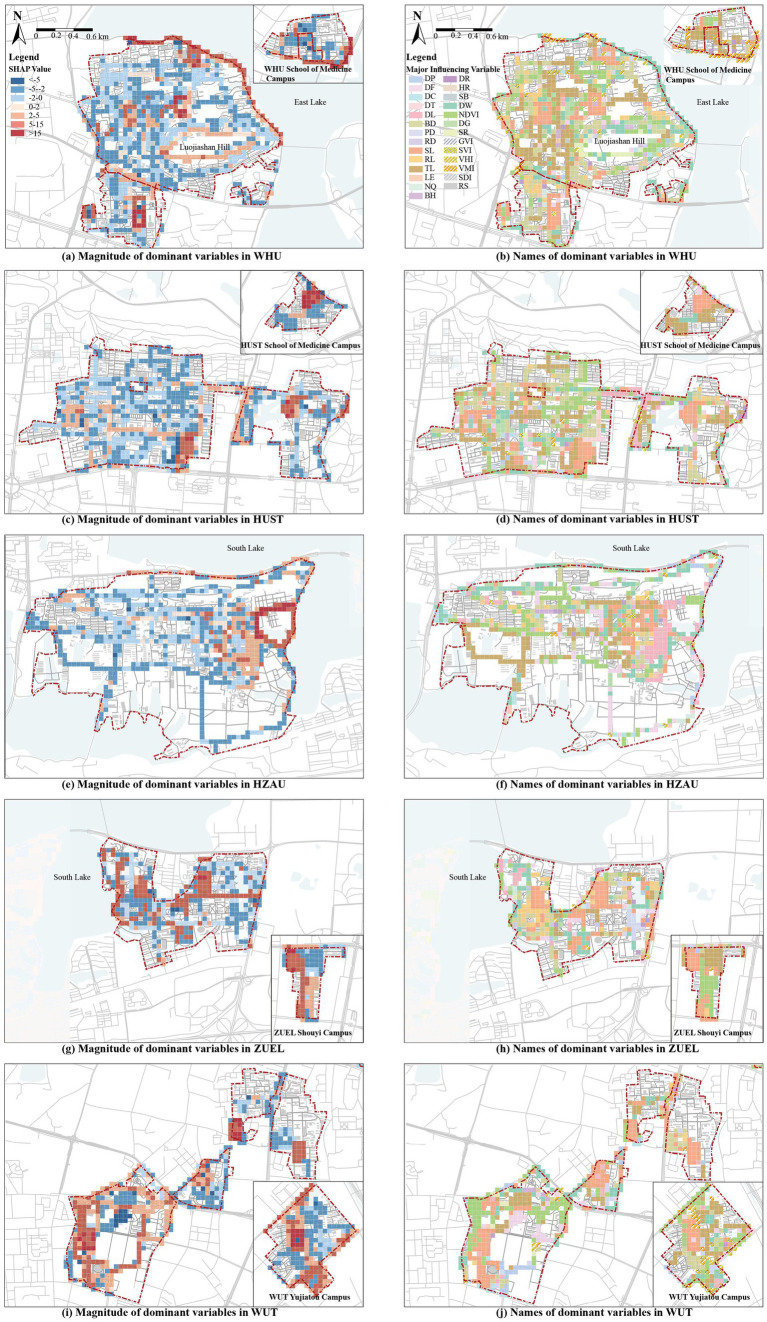
Distribution of dominant factors of local impacts obtained from the SHAP model. **(a)** Magnitude of dominant variables in WHU; **(b)** Names of dominant variables in WHU; **(c)** Magnitude of dominant variables in HUST; **(d)** Names of dominant variables in HUST; **(e)** Magnitude of dominant variables in HZAU; **(f)** Names of dominant variables in HZAU; **(g)** Magnitude of dominant variables in ZUEL; **(h)** Names of dominant variables in ZUEL; **(i)** Magnitude of dominant variables in WUT; **(j)** Names of dominant variables in WUT.

The relative importance (RI) of independent variables was measured using the average absolute SHAP values, which reflect the extent to which each feature influences the model’s output. After calculating the mean absolute SHAP values for all features, the variables were ranked from highest to lowest. The analysis was visualized using bar plots, beeswarm plots, and ArcGIS maps (Figures 4, 5) (47).

The relative contribution of macroscale CBE variables and microscale CBE variables to EW is 86.75 and 13.25%, respectively. This indicates that macroscale built environments variables within campuses play a dominant role in influencing exercise walking (Figure 4A). The top-ranking independent variables in terms of RI are in order, SL (8.92%), NDVI (7.69%), DW (7.40%), DF (6.50%), TL (6.18%), BD (5.99%), VHI (4.58%), LE (4.06%), RL (4.03%), DL (3.89%), NQ (3.52%), DP (3.51%), DR (3.49%), PD (3.30%), and the influence of other variables accounted for less than 3%. Among them, the influence of sports land use density is the most significant, indicating that the quantity and accessibility of sports facilities are closely linked to campus walking activities. This is consistent with previous research findings (32, 40). Similar to findings in park environments, beautiful natural landscapes and aesthetic human-made features significantly enhance walking activities (40, 52), likely due to the scenic and recreational appeal of natural features, which improves the walking experience. Particularly, Wuhan’s University campuses, with their proximity to water bodies, demonstrate strong positive impacts on walking activities, consistent with recent studies highlighting the positive effects of urban water environments on physical activity (53). The influence of residential land use proportion, educational and research land use proportion, building density, and land use diversity further supports their critical role in influencing EW within campus environments (32, 54).

Notably, CBE variables related to spatial structure and road connectivity, such as road density, route efficiency, block scale, and betweenness centrality, exhibit relatively weak influence. Similarly, DG does not show significant impact. These findings are inconsistent with current studies on urban environments, where such factors are often prominent influencers of physical activity (54–56). This divergence may stem from the low building density and distinct functional zoning within campuses, as well as differing purposes of walking activities (e.g., commuting vs. exercise walking).

Beeswarm plots illustrate the distribution of SHAP values across samples where wider areas represent a high concentration of samples, while longer extensions on the right or left indicate stronger positive or negative contributions to SHAP values, respectively. Redder hues represent higher feature values, while bluer hues represent lower values (57). From Figure 4B, variables such as SL VHI, and RS exhibit positive correlations with exercise walking. Conversely, RL, TL, and BD display negative correlations. Other variables exhibit less distinct patterns, suggesting potential complex nonlinear relationships.

To clarify spatial heterogeneity of campus CBE and provides insights into the localized impacts of different environmental features on EW, we calculated the dominant influencing variables for each sample of the SHAP model. These dominant variable values were assigned to each grid, and local explanation maps were generated in ArcGIS to visualize the influence of campus environments on exercise walking (Figure 5). Figures 5A, C, E, G, I show the magnitude of dominant variables. Red represents positive impacts on EW, while blue indicates negative impacts, with deeper colors signifying stronger effects. Figures 5B, D, F, H, J annotate the names of the dominant influencing variables.

The high-impact areas across the five university campuses align with the RI rankings are concentrated in the following six typical regions:

(1) Sports fields: All five university campuses exhibit strong positive impacts in campus sports field areas. The dominant variable in these regions is the SL and its influencing areas often spills over into adjacent grids.
(2) Residential and educational zones: Large residential and science and education zones within all campuses generally have negative impacts on exercise walking. The dominant variables in these areas are RL and TL and some grids are also influenced by BD and other variables.
(3) Proximity to large water bodies: The Lakeside campus exhibit a significant positive impact in their peripheral areas along the lakes. For instance, grids near Donghu Lake at WHU, and Nanhu Lake near HZAU and ZUEL, show strong positive impacts. The dominant variable in these areas is mostly DW, with some influence from NDVI and other factors.
(4) Major boulevards of larger campus and connecting roads between campuses: In larger campuses, the boulevard has a strong positive influence on EW in surrounding grids, such as Shizishan Boulevard at HZAU, Zhongnan Boulevard at ZUEL, and Zijing Road at HUST. These roads typically feature large trees along both sides, wide pedestrian areas, less vehicular traffic, and open sight lines. Similarly, the connecting roads between multiple campuses also show strong positive effects, such as Bayi Road between the north and south campuses of WHU, and Yuyuan Road between the east and west campuses of HUST. As major transport routes linking key functional areas within and between campuses, these roads experience higher pedestrian flows, which is reasonable. The dominant influencing factors along these roads are also more diverse.
(5) Areas Along smaller campus gates: Areas such as the southern side of WHU’s Medical School, the western side of HUST’s East Campus, and the southeastern side of WUT’s Yujiatou Campus have dominant grids influenced by VMI and VHI. These areas, located at the campus-urban interface, experience relatively high urban vitality, which positively impacts exercise walking (32, 41). In smaller campuses, the proximity to campus gates enhances accessibility and efficiency, leading to a more noticeable influence from the surrounding urban environment. This makes the areas around these gates particularly significant in shaping walking patterns.
(6) Circular Paths and Greenways: Some circular road grids within WHU, HZAU, and WUT campuses show continuous positive effects. The representative one is the forest trail around Luojia Mountain at WHU, where the most dominant variable is NDVI. Similar high-impact circular roads in campuses like HZAU and WUT, such as greenways and secondary roads, exhibit diverse dominant variables. Circular paths provide a scenic, safe, and comfortable environment for walkers, enriching the walking experience and enhancing their appeal for exercise walking (22, 40).

### 3.3 Nonlinear associations between CBE and EW

**Figure 6:**
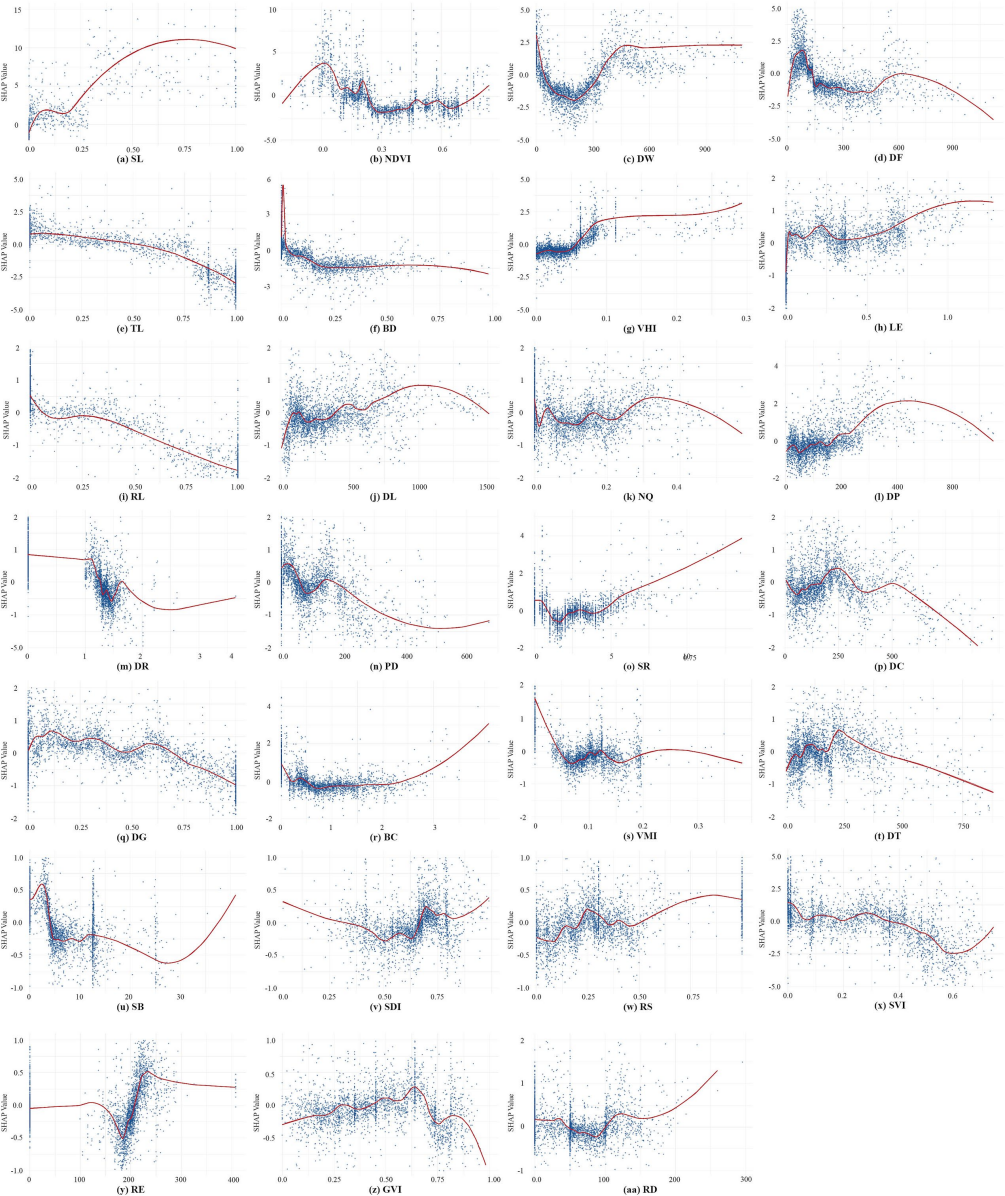
Nonlinear association between the CBE attributes and EW. **(a)** SL; **(b)** NDVI; **(c)** DW; **(d)** DF; **(e)** TL; **(f)** BD; **(g)** VHI; **(h)** LE; **(i)** RL; **(j)** DL; **(k)** NQ; **(l)** DP; **(m)** DR; **(n)** PD; **(o)** SR; **(p)** DC; **(q)** DG; **(r)** BC; **(s)** VMI; **(t)** DT; **(u)** SB; **(v)** SDI; **(w)** RS; **(x)** SVI; **(y)** RE; **(z)** GVI; **(aa)** RD.

Partial Dependence Plots (PDPs) (22) were used to visualize SHAP value dependencies, revealing nonlinear relationships between variables and EW (Figure 6). The analysis focuses on high-ranking and representative variables due to the large number of variables.

SL (Figure 6A) exhibits strong positive effects on EW when SL exceeds 0.2. The positive influence of the number of sports grounds, area, and diversity of sports facilities on the EW of walking has been generally confirmed by previous studies (22, 40, 58). However, specific studies investigating the trend of this influence remain scarce.

NDVI (Figure 6B) exhibits complex nonlinear effects on EW. The effect curve reveals two peak thresholds, one near 0 and another around 0.2, but presents a predominantly negative effect in the range between 0.25 to 0.75. Areas with an NDVI close to 0 may correspond to waterside trails, while the range around 0.2 likely represents fixed exercise locations, such as sports fields. These trends are generally aligned with prior studies on the effects of urban vegetation coverage on physical activity (21, 37), indicating that moderate vegetation may enhances walking by improving the environment, while dense vegetation may reduce openness, safety, and accessibility, thereby negatively impacting walking activity.

DW (Figure 6C) displays a “U-shaped” pattern curve within 450 meters, indicating a strong positive impact in close proximity and diminishing benefits beyond 200 meters. This finding aligns with the notion that rich waterside landscapes exert a strong attraction for walking, hereby underscoring the pivotal role of proximity to water in fostering physical activity within campus environments (59, 60).

DF (Figure 6D) also demonstrates a “U-shaped” curve pattern, with the most significant positive impact occurring within a 100-meter and diminishing returns observed beyond 500 meters. This finding underscores the active role of proximate fitness and leisure facilities in promoting walking (21, 40).

TL (Figure 6E), BD (Figure 6F), RL (Figure 6I), and PD (Figure 6N) all exert negative impacts on EW in university campuses, which is contrasted with findings from urban community studies (55, 61). Some studies have suggested that higher building density, dense population promotes walking by creating more compact urban structures with diverse functionality (55, 61). Conversely, some nonlinear studies, such as those by Yang et al., have observed negative associations between building density and physical activity (21), and have revealed that population density, beyond certain thresholds, may exert marginal effects or negative impacts (21, 62). Campuses typically feature low building density, population density and clear functional zoning, with most walkers preferring more open environments outside residential and academic areas.

VHI (Figure 6G) and VMI (Figure 6S) values are generally below 0.3, reflecting reduced pedestrian and traffic flows in campus environments compared to urban areas (20, 21). The relationship between VHI and EW demonstrates a non-uniform trend, transitioning from negative to positive around 0.06, with positive effects intensifying between 0.06 and 0.1 before leveling off. In contrast, VMI displays an inverted “U-shaped” curve pattern on EW with positive impacts observed at VMI values below 0.04, and negative impacts beyond this threshold. This suggests that low vehicle flow within campuses enhances walking safety, thereby encouraging EW (20, 52). Notably, small peaks at VMI values around 0.1 and 0.18 likely correspond to campus periphery zones where moderate motorization reflects functional diversity and higher urban vitality.

DL (Figure 6J) demonstrates positive effects on EW that increases with distance, particularly between 600 and 1,000 meters, after which the influence plateaus. This observation signifies that DL has a relatively high tolerance for walking distance in campus environment.

NQ (Figure 6K) exhibits a fluctuating impact on EW, likely due to the concentration of walking activities in fixed areas with similar closeness centrality. On the other hand, DR (Figure 6M) generally has a negative influence on EW, but exhibits a “U-shaped” trend between 1.2 and 1.7. While prior studies suggest that high closeness centrality and low detour ratios promote walking (32), this discrepancy may arise from a focus on general walking rather than walkability. Leisurely walking exercises may favor forest trails with moderate detours, aligning with findings by Peachy et al. that campus users often prefer less connected paths like cul-de-sacs or circular routes for light physical activity (52).

SR (Figure 6O) also exhibits a ““U-shaped” curve pattern, with most samples concentrated on slopes under 5 degrees. This supports the preference for rather flat terrain in walking (63, 64). However, few samples with slopes above 5 degrees also exhibit strong positive impacts, likely reflecting the preferences of fitness enthusiasts who favor challenging terrains and varied landscapes like hilly areas.

DG (Figure 6Q) demonstrates a fluctuating downward trend, with predominantly negative effects when DG exceeds 0.7. This finding contrasts with studies suggesting a positive link between green density and physical activity (56), though recent nonlinear research highlights DG benefits only within certain thresholds (62). The negative trend may reflect large green spaces on campuses serving as buffer zones or isolation areas, often excluded from active use (40), and restricted access may further limit their utility for walking (65).

### 3.4 Interaction effects between CBE variables

**Figure 7:**
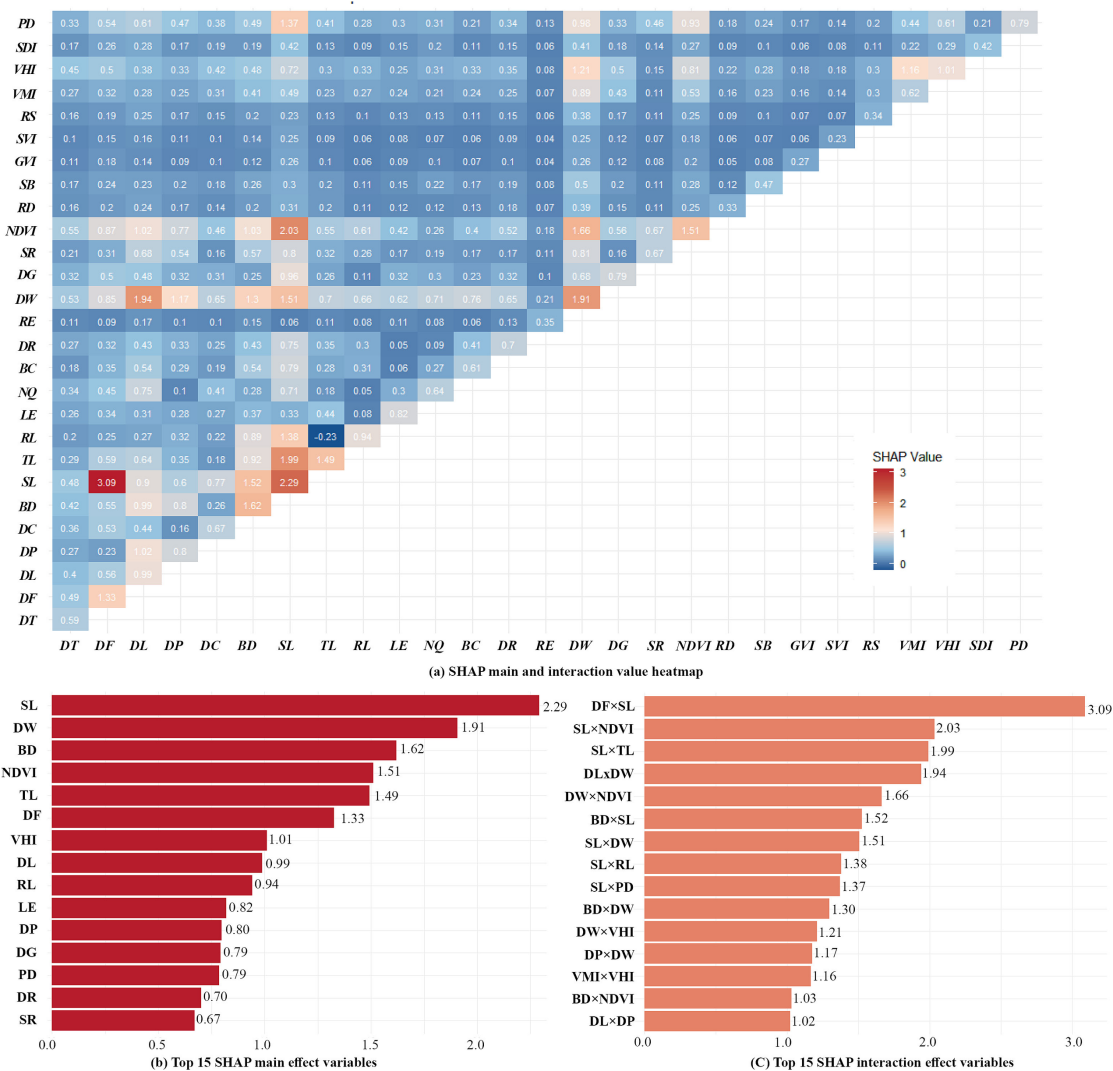
Heat maps of main and interaction effects of variables with Top 15 rankings. **(a)** SHAP main and interaction value heatmap; **(b)** TOP 15 SHAP main effect variables; **(c)** Top 15 SHAP interaction effect variables.

**Figure 8:**
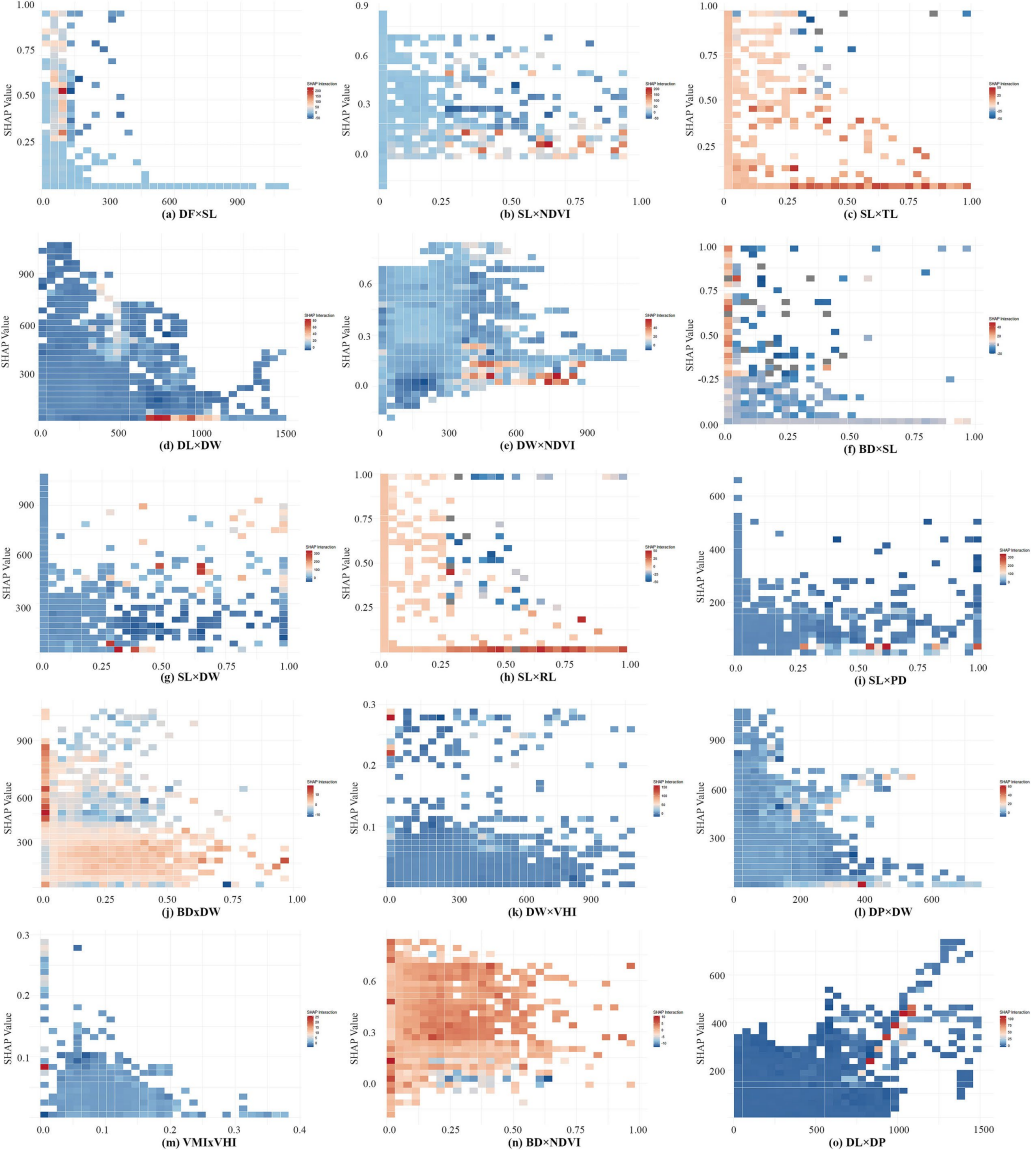
Interaction heatmap of the top 15 variable combinations. **(a)** DF×SL; **(b)** SL×NDVI; **(c)** SL×TL; **(d)** DL×DW; **(e)** DW×NDVI; **(f)** BD×SL; **(g)** SL×DW; **(h)** SL×RL; **(i)** SL×PD; **(j)** BD×DW; **(k)** DW×VHI; **(l)** DP×DW; **(m)** VMI×VHI; **(n)** BD×NDVI; **(o)** DL×DP.

To further explore interaction and synergistic effects existed among CBE variables on EW, we utilized the SHAP model’s ‘shapviz’ package to decompose each variable’s SHAP values into main effects and interaction effects, visualized through a heat map matrix (Figure 7A). Additionally, we ranked the top 15 variable combinations based on both main and interaction effects (Figures 7B,C). From the analysis results, we found most variable pairs exhibit interaction effects, with positive synergistic interactions being the dominant pattern. In some cases, these interaction effects between variables surpassed the main effects of individual variables. To enhance clarity, the interaction effect ranges of the top 15 combinations were displayed in heatmaps (Figure 8). These heatmaps reveal that extreme values within certain threshold ranges influenced overall interaction effect size, leading to uneven distributions across thresholds. A few representative variable pairs with distinct intervals were further analyzed.

DF and SL (Figure 8A) shows a strong positive interaction effect when SL exceeds 0.25 and fitness facilities are within 100 meters. In other ranges, the interaction effect is predominantly negative. This suggests that combining large sports fields and smaller fitness or leisure spaces meets diverse user needs, enhancing opportunities for EW (32, 40).

Similarly, SL and NDVI (Figure 8B) also indicate a strong positive interaction. The positive synergistic influence is more significant when the SL reaches 0.25 or more and the NDVI is below 0.15, indicating that sports grounds with open surroundings enhance EW.

When SL values is greater than 0.25, TL (Figure 8C) RL (Figure 8H) and BD (Figure 8F) around o, the positive synergistic influence is stronger. The finding aligns with the negative relationships observed in prior analyses.

DW also exhibits strong interaction effects with several variables. Among them, when DW is close to 0(waterfront areas), the positive synergistic effect on walking is stronger when scenic spot-in campus are within 600 to 1,000 meters (Figure 8D), or when VHI is greater than 0.2(Figure 8K). In contrast, other intervals show weaker positive effect. Between 400 and 900 meters from water, stronger positive synergy appears in areas with NDVI below 0.15 (Figure 8E) or low building density (Figure 8J). These areas often feature fixed exercise zones like sports fields and fitness facilities, which exert greater influence on EW than waterfront areas or dense built environments.

VMI and VHI (Figure 8M) exhibit more polarized changes in their interaction effects. A strong synergistic effect on EW when VMI is close to 0 and VHI is close to 0.09, but less pronounced effects in other ranges.

The interaction between BD and NDVI (Figure 8N) was negative in areas where BD exceeds 0.25 and NDVI approaches 0, while it is generally positive in the other regions. This indicates that, for on-campus walkers, a certain amount of vegetation cover and lower building density have a positive synergistic effect on walking. In contrast, areas with minimal vegetation and high building densities are less conducive for EW.

## 4 Discussion

This study applied multidimensional data and interpretable machine learning methods to explore the nonlinear impact mechanisms through which and how university campus building environments influence exercise walking. The finding highlights key features and areas that influence walking, and reveal their thresholds and change patterns that would promote or prohibit walking. This research not only refines our understanding of these relationships but also provides targeted insights for optimizing campus planning and design.

### 4.1 Analysis of variables’ RI

By analyzing variable importance and distribution, six types of significant campus areas were identified that notably influence exercise walking. The dominant variables in these regions are diverse, reflecting the hybrid characteristics of campus built environments that blend features of urban communities and parks. These factors make campus walking influenced by elements common to both community and park environments, while also showing distinctive characteristics. Campuses can thus be viewed a unique “neighbourhoods” within urban settings (32).

Similar to urban community environments, the macroscale built environments features in campuses play a dominant role in influencing exercise walking. This observation aligns with Alfonzo’s hierarchy of needs model (the “pyramid”) (24), where macroscale variables reflect basic needs like accessibility and safety, and micro-scale variables mostly address needs for comfort and enjoyment. Also, variables such as sports facility accessibility, building density, and land use diversity rank high in importance for campus exercise walking, mirroring trends observed in urban settings (32). However, factors like transportation connectivity, population density, road density, commercial services and transit station accessibility, which are crucial in urban neighbourhoods, have less influence within campuses.

Similar to urban parks, exercise walking in campuses is closely related to natural scenery, the availability of sports facilities, scenic spots, and trail quality, with these variables ranking highly in terms of influence (35, 40, 58). The study reveals that walkers in campuses prefer waterside and forest trails, often favoring circular walking patterns (59). However, unlike parks, campuses also exhibit strong correlations between exercise walking and land use types, building density, and other campus-specific factors that are typically absent in park environments.

### 4.2 Nonlinear effects of variables

The distinct nature of campus built environments leads to differences in how they influence exercise walking compared to typical urban environments. In urban settings, high residential land use density and street connectivity are generally associated with increased opportunities for walking or cycling. Dense, multifunctional communities provide diverse destinations, such as public spaces, parks, streets, and transit hubs, which encourage walking activities (54, 55, 66). In contrast, this study found that in campus environments residential landuse proportion, building density, and green space density generally have an overall negative impact on exercise walking. This may be attributed to the lower building density, clearly defined functional zoning, and the preference of campus walkers for specific locations, such as trails and sports fields, rather than residential or academic areas.

Additionally, the influence trends and threshold ranges of macroscale environmental variables within campuses often differ significantly from those observed in urban environments. Some variables exhibit multiple high and low thresholds that require careful attention. For instance, the NDVI shows two positive peaks near 0 and 0.2, a negative influence between 0.25 and 0.75, and a return to positive influence beyond 0.75. This nonlinear pattern is inconsistent with trends observed in urban environments (21, 24). Similarly, variables such as Proximity to Water Bodies, Detour Ratio, Slope rate, Shannon Diversity Index, Route Efficiency, and Road Density all exhibit U-shaped or V-shaped influence curves. These patterns suggest that the effects of these variables are subject to marginal effects (21, 62), where their influence either promotes or suppresses exercise walking within specific threshold ranges. Therefore, these threshold ranges should be carefully considered in campus planning and design.

Finally, microscale street environment variables also exhibit complicated nonlinear effects, with trends and thresholds differing from those in urban environments. For example, Yang et al. found that in Beijing’s urban environment, the Visual Humanization Index had a primarily positive effect on jogging when within the 0.021–0.033 range, but its influence declined beyond 0.033, possibly due to heavy non-motorized traffic restricting jogging spaces and routes (19). In contrast, this study found that in campus settings, VHI primarily exerts a positive effect beyond 0.06. This divergence likely arises because campuses have more open public spaces, where higher Visual Humanization Index does not disrupt walkers but instead attract them to vibrant active areas.

### 4.3 Interaction effects among variables

So far, Few studies have highlighted the significant synergistic effects among built environments variables in urban settings, with strong combinations often involving connectivity and accessibility-related indicators (21, 67). This study finds that within campus built environments, variables with high RI rankings, such as Sports Land Use, Proximity to Water Bodies, and NDVI, exhibit notable interactive effects when combined with other variables on EW. This phenomenon can be attributed to the unique characteristics of campuses, which combine the features of both “communities” and “parks, “offering rich facilities and natural resources. The strongest synergistic combinations primarily involve macro–macro built environments variables, followed by macro–micro and micro–micro combinations. Most combinations exhibit polarized impact ranges, indicating that their influence on exercise walking can vary significantly depending on specific spatial arrangements and thresholds.

For macro–macro combinations, the arrangement of sports fields and fitness or leisure facilities in campus planning and design is particularly important. Pairing sports fields with residential areas and lower building densities can effectively promote walking exercise. Similarly, creating and utilizing waterfront environments, adding more natural and cultural scenic spots, and integrating green landscapes with moderate building density can create a visually appealing and functional environment that supports exercise walking.

Regarding macro–micro and micro–micro combinations, the design of vibrant activity zones near water bodies, as well as human activity nodes in car-free areas, plays a positive role in fostering exercise walking. These findings align with Whyte’s classic assertion that “people are the greatest attraction in public spaces” (68). They also underscore that walking in campuses serves dual purposes, functioning both as an exercise activity and a means of leisure, relaxation, and socialization.

### 4.4 Limitations

This study has several limitations. First, future research should incorporate more recent data and integrate sociodemographic information to build a more comprehensive framework. Additionally, the relatively small sample size and skewed distribution of walking trajectory data across subsets may have impacted the model’s generalization ability. Second, the study focused solely on walking frequency as the dependent variable, without considering variations in walking patterns (e.g., circular vs. linear) or behavioral differences across weekdays, weekends, and seasons. Including seasonal differences could provide deeper insights into walking behaviors in different contexts. Finally, as the sample was limited to university campuses in Wuhan, the findings may not be directly generalizable to other campuses. To address these limitations, future studies should expand the scope, refine data selection to ensure balanced datasets, incorporate diverse sociodemographic data and different walking patterns, and develope a more comprehensive research framework. In the future, we could extend our study to predict exercise walking across different campus environments to inform urban planning and improve campus health initiatives, while assessing the model robustness of the model for different scenarios.

## 5 Conclusions and practical implications

This study introduces a framework to explore the nonlinear impact of campus built environments on exercise walking. It identifies critical thresholds of environmental factors and verifies the synergistic effects of variable interactions on exercise walking. The results highlight that university campuses uniquely integrate features of urban communities and parks, requiring tailored interventions.

In campus design and planning, leveraging the high-impact areas, such as sports fields, recreational facilities, and natural landscape resources, is essential for enhancing walking activities. Particular attention should be paid to the nonlinear influence intervals and critical thresholds of built environments variables on exercise walking, with targeted interventions implemented based on the distinct characteristics of different campus types. For example, leveraging the superior natural conditions of lakeside campuses, enhancing major boulevards in larger campuses, improving areas around gates in smaller campuses, and optimizing connecting roads between multi-campus systems can help address the limitations of one-size-fits-all approaches. Campus design should also align with walkers’ psychological preferences by incorporating natural and cultural scenic spots, thoughtfully designing green landscapes and built environments, and enhancing the visual richness and aesthetic appeal of campus spaces. Expanding the area and diversity of sports facilities, adding various sizes of sports fields, improving land use and functional diversity, and constructing circular trails and secondary road networks can significantly promote exercise walking and enrich the walking experience. Additionally, integrating rest areas and organizing activities in key locations can foster social interaction and increase the vibrancy of campus spaces. Lastly, particular attention should be given to the scenic value of waterfront areas within campuses. Developing well-designed waterfront walking trails can greatly enhance the walking experience, creating more attractive and engaging environments for exercise walking. This study provides valuable scientific evidence for optimizing campus planning and design, offering effective strategies to create inclusive, healthy, and sustainable campus environments.

## Data Availability

The raw data supporting the conclusions of this article will be made available by the authors, without undue reservation.
