# Supplementary figures and images for: Exploring nonlinear and interaction effects of urban campus built environments on exercise walking using crowdsourced data
Source: Front Public Health. 2025 Jan 30;13:1549786. doi: 10.3389/fpubh.2025.1549786 (PMC11821617; doi:10.3389/fpubh.2025.1549786)

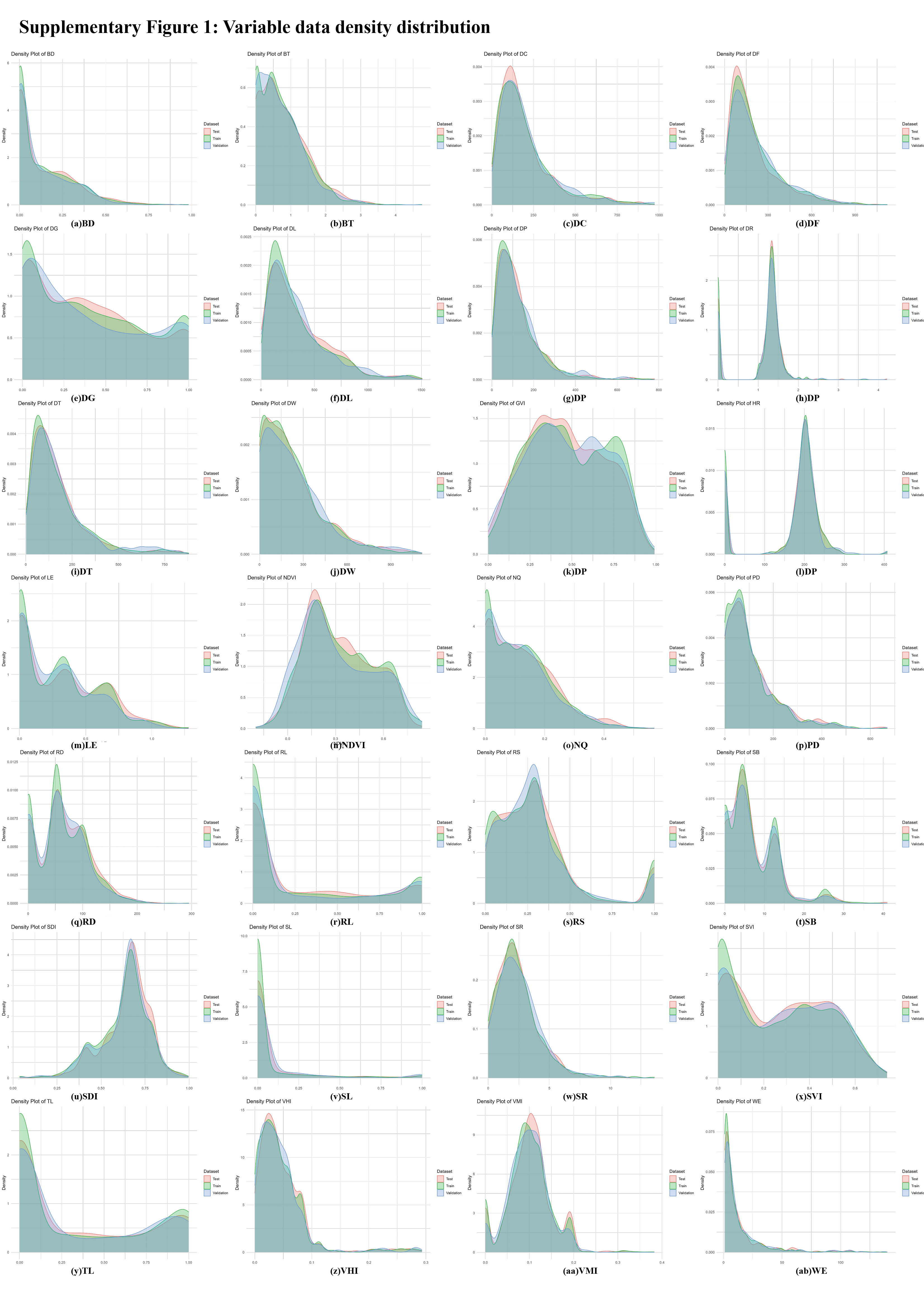

Supplement: Supplementary file 1 [file Image_1.jpg]

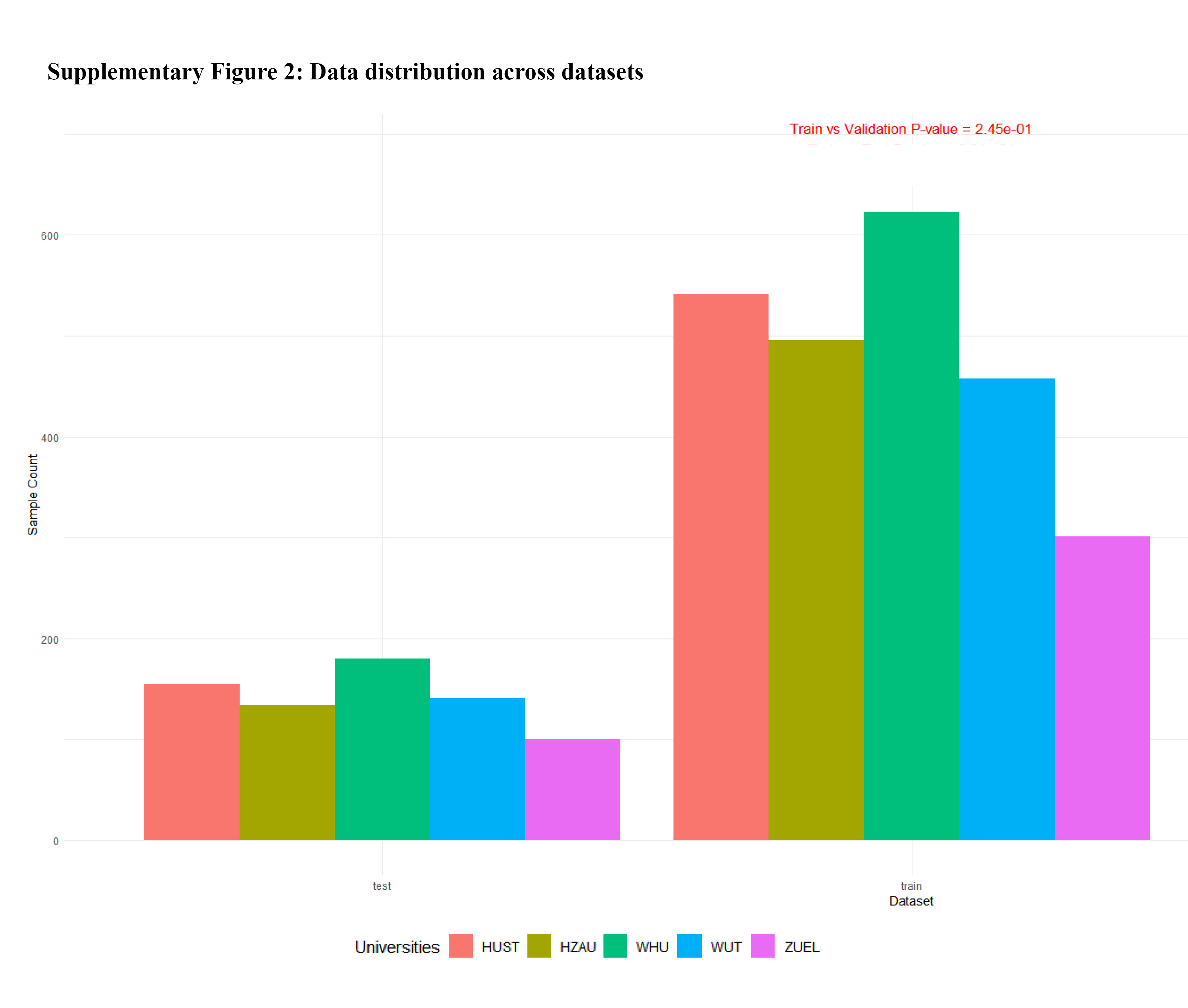

Supplement: Supplementary file 2 [file Image_2.jpg]
