# Supplementary material for: Exploring nonlinear and interaction effects of urban campus built environments on exercise walking using crowdsourced data
Source: Front Public Health. 2025 Jan 30;13:1549786. doi: 10.3389/fpubh.2025.1549786 (PMC11821617; doi:10.3389/fpubh.2025.1549786)
Supplement: Supplementary file 3 [file Table_1.docx]

**Supplementary table 1: Data descriptive statistics**

| Variable | Statistic | Test Set | Training Set | Validation Set |
| --- | --- | --- | --- | --- |
| BD | Max | 0.802893354 | 0.982234148 | 0.978637044 |
| BD | Mean | 0.129721029421053 | 0.127390860017678 | 0.149555159527985 |
| BD | Median | 0.053217436 | 0.057560276 | 0.091398757 |
| BD | Min | 0 | 0 | 0 |
| BD | Q1 | 0 | 0 | 0 |
| BD | Q3 | 0.236993316 | 0.225179213 | 0.250027269 |
| BD | SD | 0.16283218456837 | 0.15820174820448 | 0.176638238879185 |
| BT | Max | 4.754657 | 4.092568 | 2.840261 |
| BT | Mean | 0.745238336466165 | 0.754513909200482 | 0.750580917910448 |
| BT | Median | 0.617855 | 0.632702 | 0.627634 |
| BT | Min | 0 | 0 | 0 |
| BT | Q1 | 0.234585 | 0.247316 | 0.26766475 |
| BT | Q3 | 1.1310695 | 1.142941 | 1.11101425 |
| BT | SD | 0.653439194657929 | 0.657670004004298 | 0.618804851913942 |
| DC | Max | 916.12383 | 968.52818 | 974.337492 |
| DC | Mean | 206.790397092105 | 199.140441797509 | 203.44826368097 |
| DC | Median | 156.175844 | 154.011514 | 148.219377 |
| DC | Min | 5.305797 | 2.261244 | 1.440837 |
| DC | Q1 | 85.46327775 | 85.987974 | 92.85000425 |
| DC | Q3 | 266.1613013 | 256.662162 | 262.3456073 |
| DC | SD | 172.515205253713 | 164.233002224915 | 172.764197458896 |
| DF | Max | 1034.14673 | 1077.525788 | 1121.456279 |
| DF | Mean | 209.399946050752 | 210.766264314182 | 207.943598289179 |
| DF | Median | 151.672834 | 157.599005 | 163.763643 |
| DF | Min | 4.83334 | 1.898805 | 3.861368 |
| DF | Q1 | 85.58396975 | 86.671009 | 92.04104375 |
| DF | Q3 | 285.042957 | 283.497076 | 269.007781 |
| DF | SD | 175.281814601457 | 170.36340317271 | 166.538977407148 |
| DG | Max | 1 | 1 | 1 |
| DG | Mean | 0.403102846631579 | 0.397273610928084 | 0.401335978951493 |
| DG | Median | 0.344625 | 0.345294 | 0.323809 |
| DG | Min | 0 | 0 | 0 |
| DG | Q1 | 0.05488525 | 0.079099 | 0.06562225 |
| DG | Q3 | 0.674807 | 0.65636 | 0.6968965 |
| DG | SD | 0.344110462649194 | 0.332966937485665 | 0.346465168457202 |
| DL | Max | 1464.294837 | 1509.265666 | 1479.176749 |
| DL | Mean | 355.259911426692 | 335.280124073122 | 330.261320940299 |
| DL | Median | 289.701878 | 248.67671 | 243.139673 |
| DL | Min | 8.264808 | 3.353325 | 6.564023 |
| DL | Q1 | 142.8285495 | 132.203991 | 129.4995193 |
| DL | Q3 | 496.141703 | 463.193875 | 445.6423638 |
| DL | SD | 272.884294381103 | 275.094390057916 | 278.664345094703 |
| DP | Max | 779.592422 | 750.025764 | 742.592782 |
| DP | Mean | 121.843449725564 | 122.412173160305 | 118.505957149254 |
| DP | Median | 94.274802 | 96.793627 | 91.2655115 |
| DP | Min | 4.261125 | 0.442866 | 0.78484 |
| DP | Q1 | 48.71137225 | 52.038842 | 47.122041 |
| DP | Q3 | 157.9645368 | 162.310169 | 153.1129653 |
| DP | SD | 107.073446369722 | 101.546885184464 | 103.946669429284 |
| DR | Max | 4.217105 | 3.64533 | 2.61419 |
| DR | Mean | 1.11515931015038 | 1.11451465689032 | 1.15356141044776 |
| DR | Median | 1.310593 | 1.317414 | 1.326575 |
| DR | Min | 0 | 0 | 0 |
| DR | Q1 | 1.1433125 | 1.16367 | 1.2084525 |
| DR | Q3 | 1.40593825 | 1.41081 | 1.41666475 |
| DR | SD | 0.576269315396501 | 0.554965466044118 | 0.525555822647458 |
| DT | Max | 833.628523 | 880.522337 | 824.900414 |
| DT | Mean | 172.945279129699 | 175.100952903174 | 167.620309975746 |
| DT | Median | 133.7118495 | 129.331224 | 132.286248 |
| DT | Min | 7.301426 | 2.171656 | 4.03919 |
| DT | Q1 | 73.5318095 | 70.229715 | 60.16081425 |
| DT | Q3 | 226.0205175 | 226.719919 | 215.1145258 |
| DT | SD | 145.136053908998 | 154.498694917807 | 146.338516866094 |
| DW | Max | 1115.015238 | 1093.80044 | 1054.520258 |
| DW | Mean | 233.239792704887 | 221.198056028124 | 232.596205854478 |
| DW | Median | 176.2511095 | 173.924466 | 177.013254 |
| DW | Min | 0 | 0 | 0 |
| DW | Q1 | 75.20359675 | 69.513635 | 65.974915 |
| DW | Q3 | 321.7488335 | 314.410415 | 329.7254048 |
| DW | SD | 217.840421952868 | 199.826575350749 | 214.793748904525 |
| GVI | Max | 0.930407 | 0.992462 | 0.918434 |
| GVI | Mean | 0.462654870300752 | 0.477047778625954 | 0.46315096641791 |
| GVI | Median | 0.451173 | 0.4512 | 0.4512 |
| GVI | Min | 0.001635 | 0 | 0 |
| GVI | Q1 | 0.2856355 | 0.281399 | 0.27938675 |
| GVI | Q3 | 0.639446 | 0.680522 | 0.64119925 |
| GVI | SD | 0.227519658087524 | 0.234947447908117 | 0.235645567259725 |
| HR | Max | 408.106995 | 408.106995 | 408.106995 |
| HR | Mean | 168.918942048872 | 167.518366094415 | 173.036272009328 |
| HR | Median | 198.336998 | 198.389832 | 198.8056535 |
| HR | Min | 0 | 0 | 0 |
| HR | Q1 | 174.6677508 | 172.789001 | 173.038126 |
| HR | Q3 | 214.4727518 | 213.248001 | 213.8199063 |
| HR | SD | 85.7738120732659 | 83.9148768635334 | 80.4197687780742 |
| LE | Max | 1.133485 | 1.276093 | 1.27872 |
| LE | Mean | 0.295764516917293 | 0.29297219887505 | 0.284025076492537 |
| LE | Median | 0.2707915 | 0.257198 | 0.237227 |
| LE | Min | 0 | 0 | 0 |
| LE | Q1 | 0 | 0.000714 | 0 |
| LE | Q3 | 0.51133725 | 0.498327 | 0.49169925 |
| LE | SD | 0.29024001318596 | 0.282949573404932 | 0.282178797430672 |
| NDVI | Max | 0.8411 | 0.8411 | 0.8411 |
| NDVI | Mean | 0.318650187969925 | 0.320074045801527 | 0.335131529850746 |
| NDVI | Median | 0.27495 | 0.2807 | 0.3125 |
| NDVI | Min | -0.1575 | -0.1997 | -0.1575 |
| NDVI | Q1 | 0.154575 | 0.1547 | 0.16775 |
| NDVI | Q3 | 0.47525 | 0.4754 | 0.496425 |
| NDVI | SD | 0.214310524614207 | 0.21396075144875 | 0.208847686560279 |
| NQ | Max | 0.454784 | 0.569845 | 0.451937 |
| NQ | Mean | 0.112933979323308 | 0.117345454399357 | 0.118411044776119 |
| NQ | Median | 0.1019545 | 0.099317 | 0.10147 |
| NQ | Min | 0 | 0 | 0 |
| NQ | Q1 | 0.0188735 | 0.020893 | 0.0250235 |
| NQ | Q3 | 0.180958 | 0.182427 | 0.18298375 |
| NQ | SD | 0.0962592228208251 | 0.105441095354789 | 0.101600870770856 |
| PD | Max | 645.670003 | 670.523987 | 530.036987 |
| PD | Mean | 111.070067296992 | 105.368860061069 | 91.2169700373134 |
| PD | Median | 74.164902 | 76.842102 | 64.6756735 |
| PD | Min | 0 | 0 | 0 |
| PD | Q1 | 38.976989 | 34.53595 | 27.5359 |
| PD | Q3 | 149.996624 | 145.009995 | 119.702367 |
| PD | SD | 109.611640233564 | 100.086542034543 | 93.9678092240415 |
| RD | Max | 201.372992 | 295.267751 | 217.207957 |
| RD | Mean | 63.5164615902256 | 61.5474591209321 | 61.3685178955224 |
| RD | Median | 57.3382105 | 54.540766 | 53.004418 |
| RD | Min | 0 | 0 | 0 |
| RD | Q1 | 35.85679875 | 23.067102 | 35.315253 |
| RD | Q3 | 98.89753925 | 95.490645 | 90.3108535 |
| RD | SD | 44.0060780820067 | 46.3349586191863 | 43.4281457171212 |
| RL | Max | 1 | 1 | 1 |
| RL | Mean | 0.230154614661654 | 0.234725272398554 | 0.220642164179104 |
| RL | Median | 0 | 0 | 0 |
| RL | Min | 0 | 0 | 0 |
| RL | Q1 | 0 | 0 | 0 |
| RL | Q3 | 0.41123025 | 0.413229 | 0.40838525 |
| RL | SD | 0.361570241567634 | 0.365134395297391 | 0.359861723453385 |
| RS | Max | 1 | 1 | 1 |
| RS | Mean | 0.289168145105263 | 0.299535981691041 | 0.286901302735075 |
| RS | Median | 0.267102258 | 0.264467703 | 0.262866847 |
| RS | Min | 0 | 0 | 0 |
| RS | Q1 | 0.133936688 | 0.126915285 | 0.129929129 |
| RS | Q3 | 0.363235784 | 0.373939961 | 0.361933157 |
| RS | SD | 0.232189105007344 | 0.252847142450569 | 0.235705591451626 |
| SB | Max | 40.876607 | 41.040114 | 33.258692 |
| SB | Mean | 6.93719495488722 | 6.71082354600241 | 7.406235625 |
| SB | Median | 5.0217185 | 4.958457 | 5.2357085 |
| SB | Min | 0 | 0 | 0 |
| SB | Q1 | 3.13760075 | 2.861427 | 3.25030575 |
| SB | Q3 | 12.32216125 | 11.528197 | 12.5197405 |
| SB | SD | 6.28481418501068 | 6.23601184809742 | 6.63952432008999 |
| SDI | Max | 0.996431868 | 0.980953326 | 0.996774374 |
| SDI | Mean | 0.643237408954887 | 0.628922605924066 | 0.641394941972015 |
| SDI | Median | 0.664140424 | 0.662000107 | 0.662701294 |
| SDI | Min | 0.16210415 | 0.037358796 | 0.04616229 |
| SDI | Q1 | 0.577872008 | 0.550027412 | 0.565686783 |
| SDI | Q3 | 0.728195437 | 0.716331142 | 0.722632519 |
| SDI | SD | 0.131800655201383 | 0.140607471978703 | 0.139025012705994 |
| SL | Max | 1 | 1 | 1 |
| SL | Mean | 0.067361279806391 | 0.0643424910932101 | 0.0605436229160448 |
| SL | Median | 0 | 0 | 0 |
| SL | Min | 0 | 0 | 0 |
| SL | Q1 | 0 | 0 | 0 |
| SL | Q3 | 0 | 0 | 0 |
| SL | SD | 0.192711128769642 | 0.189023648111115 | 0.190264043951566 |
| SR | Max | 11 | 13.6 | 10.666667 |
| SR | Mean | 2.3580282612782 | 2.47523659019687 | 2.33307938246269 |
| SR | Median | 2 | 2.142857 | 2 |
| SR | Min | 0 | 0 | 0 |
| SR | Q1 | 1 | 1.25 | 1 |
| SR | Q3 | 3.2 | 3.333333 | 3.2125 |
| SR | SD | 1.74927123534753 | 1.80791142817319 | 1.68072931304163 |
| SVI | Max | 0.731754 | 0.737301 | 0.73982 |
| SVI | Mean | 0.273448001558271 | 0.263492879926477 | 0.259173513363806 |
| SVI | Median | 0.282545 | 0.254548 | 0.249869 |
| SVI | Min | 0 | 0 | 0 |
| SVI | Q1 | 0.084083808 | 0.051524 | 0.04717425 |
| SVI | Q3 | 0.4473875 | 0.444739 | 0.44231775 |
| SVI | SD | 0.206468202951255 | 0.211917524623386 | 0.211782077374898 |
| TL | Max | 1 | 1 | 1 |
| TL | Mean | 0.309253281954887 | 0.311084959823222 | 0.356633729477612 |
| TL | Median | 0.0105155 | 0.01735 | 0.091718 |
| TL | Min | 0 | 0 | 0 |
| TL | Q1 | 0 | 0 | 0 |
| TL | Q3 | 0.73112275 | 0.707272 | 0.82494225 |
| TL | SD | 0.391323811733979 | 0.390814165551007 | 0.40823037084815 |
| VHI | Max | 0.274320081 | 0.293061849 | 0.290916 |
| VHI | Mean | 0.0436394361766917 | 0.0458722275456006 | 0.0447073003097015 |
| VHI | Median | 0.035787 | 0.035552 | 0.0362015 |
| VHI | Min | 0 | 0 | 0 |
| VHI | Q1 | 0.019308816 | 0.017965 | 0.01832625 |
| VHI | Q3 | 0.0574235 | 0.062067 | 0.0583805 |
| VHI | SD | 0.0397486620970622 | 0.0453648587227531 | 0.0447812918694155 |
| VMI | Max | 0.323631 | 0.382618 | 0.368246 |
| VMI | Mean | 0.100177069389098 | 0.096791143882282 | 0.101553258503731 |
| VMI | Median | 0.100212 | 0.097038 | 0.0983185 |
| VMI | Min | 0 | 0 | 0 |
| VMI | Q1 | 0.07368425 | 0.070006 | 0.06850075 |
| VMI | Q3 | 0.123403 | 0.123403 | 0.12750475 |
| VMI | SD | 0.0470393314316754 | 0.0486580295987385 | 0.051825256961934 |
| WE | Max | 126 | 133 | 140 |
| WE | Mean | 12.9718045112782 | 12.940940136601 | 12.1921641791045 |
| WE | Median | 6 | 6 | 6 |
| WE | Min | 1 | 1 | 1 |
| WE | Q1 | 2 | 2 | 2 |
| WE | Q3 | 15 | 15 | 13 |
| WE | SD | 19.061333114181 | 18.6641961500108 | 18.501791127719 |
