# Supplementary material for: Exploring nonlinear and interaction effects of urban campus built environments on exercise walking using crowdsourced data
Source: Front Public Health. 2025 Jan 30;13:1549786. doi: 10.3389/fpubh.2025.1549786 (PMC11821617; doi:10.3389/fpubh.2025.1549786)
Supplement: Supplementary file 4 [file Table_2.docx]

**Supplementary table 2: Data Kolmogorov-Smirnov test results**

| Variable | Dataset_Comparison | KS_Statistic | P_Value |
| --- | --- | --- | --- |
| DT | Train vs Validation | 0.0476242088760718 | 0.382720977222099 |
| DT | Train vs Test | 0.0350250261901991 | 0.510984081276139 |
| DT | Validation vs Test | 0.0469010786027642 | 0.600555322732666 |
| DF | Train vs Validation | 0.0310168966651828 | 0.876076681346434 |
| DF | Train vs Test | 0.0623734140379467 | 0.0279276201784168 |
| DF | Validation vs Test | 0.0814064716165848 | 0.0583710497354281 |
| DL | Train vs Validation | 0.0416581183347204 | 0.554512293485284 |
| DL | Train vs Test | 0.0473402397858223 | 0.170681129312145 |
| DL | Validation vs Test | 0.0454992248573407 | 0.63902402699807 |
| DP | Train vs Validation | 0.0488396849052587 | 0.352068725697337 |
| DP | Train vs Test | 0.0279362123152136 | 0.784956952996705 |
| DP | Validation vs Test | 0.0578883134874823 | 0.333344004133582 |
| DC | Train vs Validation | 0.0509483809781872 | 0.302829288722763 |
| DC | Train vs Test | 0.0354033290653009 | 0.496997872406629 |
| DC | Validation vs Test | 0.0672362041099054 | 0.179356273022876 |
| BD | Train vs Validation | 0.0542077147889369 | 0.236548737513656 |
| BD | Train vs Test | 0.0208648585729252 | 0.970628973492303 |
| BD | Validation vs Test | 0.0579971633077151 | 0.331140923356146 |
| SL | Train vs Validation | 0.0330820446319701 | 0.821864323373539 |
| SL | Train vs Test | 0.028134093819113 | 0.77767371886324 |
| SL | Validation vs Test | 0.0172774351024178 | 0.99999835115426 |
| TL | Train vs Validation | 0.0233998490332321 | 0.988676079541064 |
| TL | Train vs Test | 0.0188627633570016 | 0.989754303608879 |
| TL | Validation vs Test | 0.0321997559125243 | 0.945016469476897 |
| RL | Train vs Validation | 0.033983006561248 | 0.795989040682594 |
| RL | Train vs Test | 0.0240018624141544 | 0.909875933247647 |
| RL | Validation vs Test | 0.0560642543787314 | 0.371719603629493 |
| LE | Train vs Validation | 0.0536928794007781 | 0.24622983894452 |
| LE | Train vs Test | 0.0424572226748923 | 0.275697957121104 |
| LE | Validation vs Test | 0.0801200646501962 | 0.0652104674406074 |
| NQ | Train vs Validation | 0.0412826368862137 | 0.566265446506029 |
| NQ | Train vs Test | 0.0338610173437318 | 0.554999582108748 |
| NQ | Validation vs Test | 0.0584226671504436 | 0.322623594152856 |
| BT | Train vs Validation | 0.0508854781582054 | 0.30422503587238 |
| BT | Train vs Test | 0.0356244907461296 | 0.488902286343426 |
| BT | Validation vs Test | 0.0617541313454497 | 0.2611135688185 |
| DR | Train vs Validation | 0.032730756575764 | 0.83162445619085 |
| DR | Train vs Test | 0.0212373414037947 | 0.965470560748983 |
| DR | Validation vs Test | 0.0410495761453969 | 0.759929507808198 |
| HR | Train vs Validation | 0.0399316778601429 | 0.609092206799762 |
| HR | Train vs Test | 0.0233150971947387 | 0.926520181086739 |
| HR | Validation vs Test | 0.0380677507668964 | 0.834437765173083 |
| DW | Train vs Validation | 0.0381878181431087 | 0.665045904203329 |
| DW | Train vs Test | 0.0265976021417763 | 0.832197486491043 |
| DW | Validation vs Test | 0.0459313256588713 | 0.627136244682299 |
| DG | Train vs Validation | 0.0557057696401959 | 0.210004599675397 |
| DG | Train vs Test | 0.0376324060062857 | 0.418472206204627 |
| DG | Validation vs Test | 0.0707424877131642 | 0.13862420875402 |
| SR | Train vs Validation | 0.0514351520312772 | 0.292180499095162 |
| SR | Train vs Test | 0.0215108834827145 | 0.961334895745724 |
| SR | Validation vs Test | 0.0657980670910711 | 0.19857029820982 |
| NDVI | Train vs Validation | 0.0948206785762673 | 0.00292773461692651 |
| NDVI | Train vs Test | 0.0406646490513328 | 0.324126370083035 |
| NDVI | Validation vs Test | 0.0740376686347594 | 0.107504094425078 |
| RD | Train vs Validation | 0.0519025683705267 | 0.282208378524856 |
| RD | Train vs Test | 0.0487894307996741 | 0.146507820071304 |
| RD | Validation vs Test | 0.0791437147475014 | 0.070847793974704 |
| SB | Train vs Validation | 0.0364158940910059 | 0.721571122707268 |
| SB | Train vs Test | 0.0234722383890118 | 0.922872100374185 |
| SB | Validation vs Test | 0.044427219052017 | 0.6685441316637 |
| GVI | Train vs Validation | 0.0430826252733853 | 0.510723446783844 |
| GVI | Train vs Test | 0.0611046443952975 | 0.0331690003663713 |
| GVI | Validation vs Test | 0.0647326582445493 | 0.213804255677821 |
| SVI | Train vs Validation | 0.0451216443764879 | 0.450805359521442 |
| SVI | Train vs Test | 0.0660051216389244 | 0.0167390578775574 |
| SVI | Validation vs Test | 0.0696968697430484 | 0.149903013397861 |
| RS | Train vs Validation | 0.0487264598292914 | 0.354854726976551 |
| RS | Train vs Test | 0.0357758118961704 | 0.483398994441959 |
| RS | Validation vs Test | 0.0625490648810898 | 0.247791769177568 |
| VMI | Train vs Validation | 0.0351026767569242 | 0.762414992405395 |
| VMI | Train vs Test | 0.0616284483762076 | 0.0309086245001565 |
| VMI | Validation vs Test | 0.0561829996371672 | 0.369138380966916 |
| VHI | Train vs Validation | 0.0375810478642073 | 0.684506470757795 |
| VHI | Train vs Test | 0.0454778256314748 | 0.206253648036991 |
| VHI | Validation vs Test | 0.0549988455322096 | 0.395385991018186 |
| SDI | Train vs Validation | 0.0572396307120599 | 0.185255420041508 |
| SDI | Train vs Test | 0.0862763357001513 | 0.000564752063576796 |
| SDI | Validation vs Test | 0.0769799122604479 | 0.0848305017938158 |
| PD | Train vs Validation | 0.0315617318597945 | 0.862561340014205 |
| PD | Train vs Test | 0.0406821091840297 | 0.323628021111316 |
| PD | Validation vs Test | 0.0372794141900583 | 0.852502397562574 |
| WE | Train vs Validation | 0.0399123231463023 | 0.609710566284793 |
| WE | Train vs Test | 0.0287160982423466 | 0.75589558907727 |
| WE | Validation vs Test | 0.0422436256885576 | 0.728139383477488 |
